# Supplementary material for: Platelet-rich plasma for immature post-traumatic scars and early keloids: A scoping review
Source: PLoS One. 2026 Apr 6;21(4):e0345754. doi: 10.1371/journal.pone.0345754 (PMC13052873; doi:10.1371/journal.pone.0345754)
Supplement: S2 Text — (DOCX) [file pone.0345754.s003.docx]

**S2 Text. Completed risk of bias assessment questionnaires.**

# JBI Critical Appraisal Checklist for Case Reports – Ruiz et al., 2024

| **Question** | **Yes** | **No** | **Unclear** | **Not applicable** |
| --- | --- | --- | --- | --- |
| 1. Were patient’s demographic characteristics clearly described? | ☑ | ☐ | ☐ | ☐ |
| 2. Was the patient’s history clearly described and presented as a timeline? | ☑ | ☐ | ☐ | ☐ |
| 3. Was the current clinical condition of the patient on presentation clearly described? | ☑ | ☐ | ☐ | ☐ |
| 4. Were diagnostic tests or assessment methods and the results clearly described? | ☑ | ☐ | ☐ | ☐ |
| 5. Was the intervention(s) or treatment procedure(s) clearly described? | ☑ | ☐ | ☐ | ☐ |
| 6. Was the post-intervention clinical condition clearly described? | ☑ | ☐ | ☐ | ☐ |
| 7. Were adverse events (harms) or unanticipated events identified and described? | ☐ | ☑ | ☐ | ☐ |
| 8. Does the case report provide takeaway lessons? | ☑ | ☐ | ☐ | ☐ |

**Reviewer:** Alessandro Alvarado and Virgilio Blandon
**Date:** January 6th, 2026
**Record:** Aura Ruiz et al., 2024 – *Early intervention in scar management and cutaneous burns with autologous platelet-rich plasma*

This table summarizes the critical appraisal of the case report (Aura Ruiz et al., 2024) using the Joanna Briggs Institute (JBI) checklist for case reports. For each domain, “Yes” indicates the criterion was fully addressed, “No” indicates it was not, “Unclear” indicates insufficient information, and “Not applicable” applies when the criterion does not pertain to the study. The checklist evaluates patient demographics, history, clinical presentation, diagnostic assessments, interventions, post-intervention outcomes, adverse events, and clinical lessons.

**Comments:** The case report clearly describes patient demographics, history, clinical condition, diagnostic assessments, and the intervention using autologous platelet-rich plasma (APRP), including preparation, activation, and application details. Outcomes were systematically assessed using POSAS and VSS scales by both internal and external evaluators. Adverse events were not systematically reported; although potential skin burns were mentioned in the introduction, no harms were documented during follow-up. The report provides clear clinical lessons for APRP use in second-degree burns and highlights gaps in standardized protocols and long-term safety evidence.

# JBI Critical Appraisal of Case Series – Kim et al., 2024

| **Question** | **Yes** | **No** | **Unclear** | **Not applicable** |
| --- | --- | --- | --- | --- |
| 1. Were there clear criteria for inclusion in the case series? | ☑ | ☐ | ☐ | ☐ |
| 2. Was the condition measured in a standard, reliable way for all participants? | ☐ | ☐ | ☑ | ☐ |
| 3. Were valid methods used for identification of the condition for all participants? | ☑ | ☐ | ☐ | ☐ |
| 4. Did the case series have consecutive inclusion of participants? | ☐ | ☑ | ☐ | ☐ |
| 5. Did the case series have complete inclusion of participants? | ☐ | ☑ | ☐ | ☐ |
| 6. Was there clear reporting of the demographics of the participants? | ☑ | ☐ | ☐ | ☐ |
| 7. Was there clear reporting of clinical information of the participants? | ☑ | ☐ | ☐ | ☐ |
| 8. Were the outcomes or follow-up results of cases clearly reported? | ☑ | ☐ | ☐ | ☐ |
| 9. Was there clear reporting of the presenting site(s)/clinic(s) demographic information? | ☑ | ☐ | ☐ | ☐ |
| 10. Was statistical analysis appropriate? | ☐ | ☑ | ☐ | ☐ |

**Reviewer:** Alessandro Alvarado and Virgilio Blandon
**Date:** January 6th, 2026
**Record:** Kim et al., 2024 – Polynucleotide-based treatments for various facial scars including combat injuries

This checklist assesses methodological quality across ten key domains, including inclusion criteria, measurement and identification of the condition, participant demographics, reporting of clinical information, follow-up outcomes, site characteristics, and appropriateness of statistical analysis. Responses are coded as Yes, No, Unclear, or Not applicable. The table includes a summary comment describing strengths and limitations of the study, highlighting its descriptive nature, non-consecutive sampling, lack of standardized outcome measures, and absence of statistical analysis. This assessment is intended to inform evidence mapping and interpretation of study findings in the context of a scoping review.

**Comments:** This article is a descriptive case series documenting the efficacy of polynucleotide-based therapy for post-traumatic, post-surgical, and burn scars. Patient demographics, scar type, time since injury, and treatment details were clearly reported, and improvements were shown via pre/post photographs. Standardized or validated outcome measures were not applied, participant selection was non-consecutive, and statistical analysis was not performed. This study is included solely for evidence mapping purposes and does not allow formal risk-of-bias assessment or inferential statistics.

# (RoB 2) Albalat et al., 2022

Revised Cochrane risk-of-bias tool for randomized trials (RoB 2)

TEMPLATE FOR COMPLETION

Edited by Julian PT Higgins, Jelena Savović, Matthew J Page, Jonathan AC Sterne
on behalf of the RoB2 Development Group

**Version of 22 August 2019**

**Reviewer: Alessandro Alvarado AND Virgilio Blandon**

**Date: January 9th of 2026**

**Author / Year: Albalat et al., 2022**

**Title: Assessment of various intralesional injections in keloid: comparative analysis.**

The development of the RoB 2 tool was supported by the MRC Network of Hubs for Trials Methodology Research (MR/L004933/2- N61), with the support of the host MRC ConDuCT-II Hub (Collaboration and innovation for Difficult and Complex randomised controlled Trials In Invasive procedures - MR/K025643/1), by MRC research grant MR/M025209/1, and by a grant from The Cochrane Collaboration.


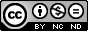


This work is licensed under a [Creative Commons Attribution-NonCommercial-NoDerivatives 4.0 International License](http://creativecommons.org/licenses/by-nc-nd/4.0/).

| **Study details**   \| **Reference** \| Albalat W, Nabil S, Khattab F. Assessment of various intralesional injections in keloid: comparative analysis. J Dermatolog Treat. 2022 Jun;33(4):2051-2056. doi: 10.1080/09546634.2021.1914307. Epub 2022 Mar 23. PMID: 33849382. \| \| --- \| --- \|   **Study design**   \| X \| Individually-randomized parallel-group trial \| \| --- \| --- \| \| □ \| Cluster-randomized parallel-group trial \| \| □ \| Individually randomized cross-over (or other matched) trial \|   **For the purposes of this assessment, the interventions being compared are defined as**   \| Experimental: \| Intralesional verapamil, intralesional 5-fluorouracil (5-FU), and intralesional platelet-rich plasma (PRP) – each compared **separately** against the control. \| Comparator: \| Intralesional triamcinolone acetonide (control group). \| \| --- \| --- \| --- \| --- \|  \| **Specify which outcome is being assessed for risk of bias** \| **Effectiveness of intralesional treatments for keloid reduction**, measured by the **Patient and Observer Scar Assessment Scale (POSAS)**. \| \| --- \| --- \|  \| **Specify the numerical result being assessed.** In case of multiple alternative analyses being presented, specify the numeric result (e.g. RR = 1.52 (95% CI 0.83 to 2.77) and/or a reference (e.g. to a table, figure or paragraph) that uniquely defines the result being assessed. \| The outcome assessed is the effectiveness of intralesional injections in treating keloids, defined as a reduction of ≥50% in the Patient and Observer Scar Assessment Scale (POSAS) score from baseline. Among the 160 patients randomized into four groups (n = 40 per group), the proportion of patients achieving this outcome was 75% in the triamcinolone acetonide group (Group A), 80% in the verapamil group (Group B), 55% in the 5-fluorouracil group (Group C), and 72% in the platelet-rich plasma group (Group D) (Results, paragraph 3; Table 3). A statistically significant difference between groups was reported (p = 0.0069). \| \| --- \| --- \|   **Is the review team’s aim for this result…?**   \| □ \| **to assess the effect of *assignment to intervention* (the ‘intention-to-treat’ effect) (x)** \| \| --- \| --- \| \| □ \| to assess the effect of *adhering to intervention* (the ‘per-protocol’ effect) \|   **If the aim is to assess the effect of *adhering to intervention***, select the deviations from intended intervention that should be addressed (at least one must be checked):  □ occurrence of non-protocol interventions  □ failures in implementing the intervention that could have affected the outcome  □ non-adherence to their assigned intervention by trial participants  **Which of the following sources were obtained to help inform the risk-of-bias assessment? (tick as many as apply)**  □ **Journal article(s) with results of the trial**  □ Trial protocol  □ Statistical analysis plan (SAP)  □ Non-commercial trial registry record (e.g. ClinicalTrials.gov record)  □ Company-owned trial registry record (e.g. GSK Clinical Study Register record)  □ “Grey literature” (e.g. unpublished thesis)  □ Conference abstract(s) about the trial  □ Regulatory document (e.g. Clinical Study Report, Drug Approval Package)  □ Research ethics application  □ Grant database summary (e.g. NIH RePORTER or Research Councils UK Gateway to Research)  □ Personal communication with trialist  □ Personal communication with the sponsor |
| --- | --- | --- | --- | --- | --- | --- | --- | --- | --- | --- | --- | --- | --- | --- | --- | --- | --- | --- | --- | --- |

Risk of bias assessment

Responses underlined in green are potential markers for low risk of bias, and responses in red are potential markers for a risk of bias. Where questions relate only to sign posts to other questions, no formatting is used.

**Domain 1: Risk of bias arising from the randomization process**

| **Signalling questions** | **Comments** | **Response options** |
| --- | --- | --- |
| **1.1 Was the allocation sequence random?** | Patients were randomly assigned to four groups using random numbers generated by Ralloc software.  The manuscript does not explicitly report concealment of the allocation sequence. | Y |
| **1.2 Was the allocation sequence concealed until participants were enrolled and assigned to interventions?** |  | NI |
| **1.3 Did baseline differences between intervention groups suggest a problem with the randomization process?** | Baseline characteristics (age, sex, Fitzpatrick skin type, keloid size) were comparable across groups. | N |
| **Risk-of-bias judgement** | Randomization was performed appropriately, but lack of clarity on allocation concealment introduces some uncertainty. | Some concerns |
| Optional: What is the predicted direction of bias arising from the randomization process? | Unclear if any systematic bias favors one group; baseline characteristics were balanced. | Unpredictable |

Domain 2: Risk of bias due to deviations from the intended interventions (*effect of assignment to intervention*)

| **Signalling questions** | **Comments** | **Response options** |
| --- | --- | --- |
| **2.1. Were participants aware of their assigned intervention during the trial?** | The study does not explicitly state whether patients were blinded. Since injections differ visibly and by procedure, participants were likely aware of their treatment.  The clinicians administering injections would necessarily know the intervention type. | PY |
| **2.2. Were carers and people delivering the interventions aware of participants' assigned intervention during the trial?** |  | Y |
| **2.3. If Y/PY/NI to 2.1 or 2.2: Were there deviations from the intended intervention that arose because of the trial context?** | No deviations from the protocol are reported; all groups received planned injections at defined intervals. | N |
| **2.4 If Y/PY to 2.3: Were these deviations likely to have affected the outcome?** | Since no deviations were reported, this is not applicable. | NA |
| **2.5. If Y/PY/NI to 2.4: Were these deviations from intended intervention balanced between groups?** |  | NA |
| **2.6 Was an appropriate analysis used to estimate the effect of assignment to intervention?** | The analysis was intention-to-treat (all randomized participants included in final analysis), and outcomes were compared appropriately. | Y |
| **2.7 If N/PN/NI to 2.6: Was there potential for a substantial impact (on the result) of the failure to analyse participants in the group to which they were randomized?** |  | NA |
| **Risk-of-bias judgement** |  | Some concerns |
| Optional: What is the predicted direction of bias due to deviations from intended interventions? | Unclear effect of awareness; potential bias cannot be confidently predicted. | Unpredictable |

Domain 2: Risk of bias due to deviations from the intended interventions (*effect of adhering to intervention*)

| **Signalling questions** | **Comments** | **Response options** |
| --- | --- | --- |
| **2.1. Were participants aware of their assigned intervention during the trial?** | Participants likely knew which injection they were receiving due to differences in procedure, volume, and sensations (e.g., burning with verapamil, ulceration with 5-FU).  Clinicians administering injections had to know which treatment was being given. | PY |
| **2.2. Were carers and people delivering the interventions aware of participants' assigned intervention during the trial?** |  | Y |
| **2.3. [If applicable:] If Y/PY/NI to 2.1 or 2.2: Were important non-protocol interventions balanced across intervention groups?** | No additional non-protocol interventions were reported; standard care and follow-up were the same for all groups. | Y |
| **2.4. [If applicable:] Were there failures in implementing the intervention that could have affected the outcome?** | All patients received the planned number of injections at the specified intervals. No deviations were reported. | N |
| **2.5. [If applicable:] Was there non-adherence to the assigned intervention regimen that could have affected participants’ outcomes?** | Adherence to the planned injection regimen was maintained in all groups. | N |
| **2.6. If N/PN/NI to 2.3, or Y/PY/NI to 2.4 or 2.5: Was an appropriate analysis used to estimate the effect of adhering to the intervention?** | Analysis included all randomized participants, comparing outcomes between groups as per intention-to-treat. | Y |
| **Risk-of-bias judgement** | Although participants and clinicians were likely aware of the intervention, there were no deviations or adherence issues affecting outcomes, and analysis was appropriate. | Some concerns |
| Optional: What is the predicted direction of bias due to deviations from intended interventions? |  | Unpredictable |

Domain 3: Missing outcome data

| **Signalling questions** | **Comments** | **Response options** |
| --- | --- | --- |
| **3.1 Were data for this outcome available for all, or nearly all, participants randomized?** | The trial randomized **160 patients (40 per group)**. The Results section reports **final POSAS scores and treatment efficacy for all four groups**, with no discrepancies in group sizes. However, the manuscript **does not explicitly report losses to follow-up, withdrawals, or a CONSORT flow diagram**.  While it *appears* that outcome data were available for nearly all randomized participants, this is **not clearly stated**. | PY |
| **3.2 If N/PN/NI to 3.1: Is there evidence that the result was not biased by missing outcome data?** | There is **no indirect evidence of bias due to missing outcome data**, such as:   - Unequal denominators across groups - Selective reporting of outcomes - Inconsistent sample sizes in analyses   Means and standard deviations for POSAS are reported for **all groups at baseline and at 24 weeks**, suggesting completeness of outcome assessment. | PY |
| **3.3 If N/PN to 3.2: Could missingness in the outcome depend on its true value?** | The study reports adverse effects in all groups, but **does not associate these with treatment discontinuation or loss to follow-up**. There is **no indication that patients with poorer outcomes were more likely to be missing from the analysis**. | PN |
| **3.4 If Y/PY/NI to 3.3: Is it likely that missingness in the outcome depended on its true value?** |  | NA |
| **Risk-of-bias judgement** | - The manuscript **fails to explicitly report the number of participants lost to follow-up or withdrawn**. - No intention-to-treat analysis or CONSORT flow diagram is provided. - However, **there is no clear evidence that missing outcome data biased the results**.   This leads to **methodological uncertainty**, not demonstrable bias. | Some concerns |
| Optional: What is the predicted direction of bias due to missing outcome data? |  | Unpredictable |

Domain 4: Risk of bias in measurement of the outcome

| **Signalling questions** | **Comments** | **Response options** |
| --- | --- | --- |
| **4.1 Was the method of measuring the outcome inappropriate?** | The **POSAS** is a **validated and widely accepted outcome measure** for scar assessment, incorporating both **patient-reported** and **observer-rated** components. Its use is appropriate for evaluating keloid response to treatment. | N |
| **4.2 Could measurement or ascertainment of the outcome have differed between intervention groups?** | The same outcome measure (POSAS) was applied **uniformly across all four groups**, at the same time points (baseline and end of treatment). There is **no indication that different instruments or assessment schedules were used** between groups. | N |
| **4.3 If N/PN/NI to 4.1 and 4.2: Were outcome assessors aware of the intervention received by study participants?** | The manuscript states that **two observers assessed outcomes**, both dermatologists, **one of whom was the treating physician**. There is **no indication that outcome assessors were blinded** to treatment allocation. Given the nature of intralesional therapies (different drugs, PRP preparation), **blinding is unlikely**. | Y |
| **4.4 If Y/PY/NI to 4.3: Could assessment of the outcome have been influenced by knowledge of intervention received?** | POSAS includes **subjective observer-rated items** (vascularity, pigmentation, thickness, pliability) and **patient-reported symptoms** (pain, pruritus, appearance). Knowledge of treatment allocation—especially when investigators believe one therapy is superior—**could influence observer scoring**, consciously or unconsciously.  Given that:   - At least one assessor was the **treating physician** - No blinding is reported - The outcome includes **subjective components** - The authors conclude superiority of one intervention   It is **likely** that outcome assessment was influenced by knowledge of treatment received. | Y |
| **4.5 If Y/PY/NI to 4.4:** **Is it likely that assessment of the outcome was influenced by knowledge of intervention received?** |  | PY |
| **Risk-of-bias judgement** | - The outcome measure itself is valid and appropriate - However, **lack of assessor blinding**, inclusion of **subjective outcome components**, and involvement of the **treating physician as an assessor** introduce a **risk of detection bias** - There is **no evidence of differential measurement between groups**, but **observer expectations could influence scores**   This does **not rise to “High risk”**, but clearly prevents a “Low risk” judgment. | Some concerns |
| Optional: What is the predicted direction of bias in measurement of the outcome? | Unblinded assessment typically biases results **toward overestimating treatment benefit**, particularly for interventions perceived as newer or more effective (e.g., verapamil or PRP). | Favours experimental |

Domain 5: Risk of bias in selection of the reported result

| **Signalling questions** | **Comments** | **Response options** |
| --- | --- | --- |
| **5.1 Were the data that produced this result analysed in accordance with a pre-specified analysis plan that was finalized before unblinded outcome data were available for analysis?** | - There is **no trial registration number** provided. - No **published or referenced protocol** is mentioned. - No **pre-specified statistical analysis plan (SAP)** is described. - The study was **unblinded**, and the analysis appears to have been conducted **after outcome data were known**.   Therefore, it is **unclear whether analyses were pre-specified or data-driven**. | NI |
| **Is the numerical result being assessed likely to have been selected, on the basis of the results, from...** |  |  |
| **5.2. ... multiple eligible outcome measurements (e.g. scales, definitions, time points) within the outcome domain?** | - POSAS allows reporting:   - Patient score alone   - Observer score alone   - Total score   - Individual domains (vascularity, pigmentation, thickness, etc.) - The authors:   - Report **only total POSAS**   - Introduce a **post-hoc dichotomization** (“>50% reduction = effective”) - Intermediate time points (e.g. after each session) are **not reported**, despite repeated treatments. - It is **unclear whether other POSAS components were measured but not reported**.   This creates a **credible risk of selective outcome measurement reporting**. | PY |
| **5.3 ... multiple eligible analyses of the data?** | Several analytic choices are evident:   - Continuous POSAS change vs categorical “effectiveness” - Group comparisons using unclear statistical tests (despite stating non-parametric data) - No adjustment for multiple comparisons across four groups   No justification is given for:   - The >50% cut-off - The specific statistical contrasts reported   This suggests **flexibility in analysis decisions**, with potential selection of analyses that favored statistically significant findings. | PY |
| **Risk-of-bias judgement** | - Absence of protocol or trial registration - No evidence of a pre-specified analysis plan - Use of **post-hoc outcome dichotomization** - Potential for **selective reporting among multiple valid POSAS summaries and analyses**   However:   - The **primary outcome domain (POSAS)** is consistent throughout - There is **no clear evidence of outright outcome switching**   Thus, the bias does **not justify “High risk”**, but **precludes “Low risk.”** | Some concerns |
| Optional: What is the predicted direction of bias due to selection of the reported result? |  | NA / Favours experimental / Favours comparator / Towards null /Away from null / Unpredictable |

Overall risk of bias

| **Risk-of-bias judgement** |  | Some concerns |
| --- | --- | --- |
| Optional: What is the overall predicted direction of bias for this outcome? |  | NA / Favours experimental / Favours comparator / Towards null /Away from null / Unpredictable |


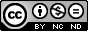


This work is licensed under a [Creative Commons Attribution-NonCommercial-NoDerivatives 4.0 International License](http://creativecommons.org/licenses/by-nc-nd/4.0/).

# (RoB 2) Neinaa et al., 2021

Revised Cochrane risk-of-bias tool for randomized trials (RoB 2)

TEMPLATE FOR COMPLETION

Edited by Julian PT Higgins, Jelena Savović, Matthew J Page, Jonathan AC Sterne
on behalf of the RoB2 Development Group

**Version of 22 August 2019**

**Reviewer: Alessandro Alvarado AND Virgilio Blandon**

**Date: January 9th of 2026**

**Author / Year: Neinaa et al., 2021**

**Title: Botulinum toxin and platelet rich plasma as innovative therapeutic modalities for keloids.**

The development of the RoB 2 tool was supported by the MRC Network of Hubs for Trials Methodology Research (MR/L004933/2- N61), with the support of the host MRC ConDuCT-II Hub (Collaboration and innovation for Difficult and Complex randomised controlled Trials In Invasive procedures - MR/K025643/1), by MRC research grant MR/M025209/1, and by a grant from The Cochrane Collaboration.


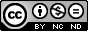


This work is licensed under a [Creative Commons Attribution-NonCommercial-NoDerivatives 4.0 International License](http://creativecommons.org/licenses/by-nc-nd/4.0/).

| **Study details**   \| **Reference** \| Neinaa YME, Elsayed TA, Mohamed DA, Elfar NN. Botulinum toxin and platelet rich plasma as innovative therapeutic modalities for keloids. Dermatol Ther. 2021 May;34(3):e14900. doi: 10.1111/dth.14900. Epub 2021 Mar 2. PMID: 33605002. \| \| --- \| --- \|   **Study design**   \| X \| Individually-randomized parallel-group trial \| \| --- \| --- \| \| □ \| Cluster-randomized parallel-group trial \| \| □ \| Individually randomized cross-over (or other matched) trial \|   **For the purposes of this assessment, the interventions being compared are defined as**   \| Experimental: \| 1. **Intralesional botulinum toxin type A (BTX-A)** 2. **Intralesional platelet-rich plasma (PRP)** \| Comparator: \| 1. **Intralesional triamcinolone acetonide (TAC)** \| \| --- \| --- \| --- \| --- \|  \| **Specify which outcome is being assessed for risk of bias** \| Change in Vancouver Scar Scale (VSS) score after treatment \| \| --- \| --- \|  \| **Specify the numerical result being assessed.** In case of multiple alternative analyses being presented, specify the numeric result (e.g. RR = 1.52 (95% CI 0.83 to 2.77) and/or a reference (e.g. to a table, figure or paragraph) that uniquely defines the result being assessed. \| **Mean percentage improvement in Vancouver Scar Scale (VSS):** BTX-A-group: 81.7% ± 19 PRP-group: 85.3% ± 18.3 TAC-group: 46.5% ± 14.3 Between-group comparison:  BTX-A vs TAC (P < .001)  PRP vs TAC (P < .001)  BTX-A vs PRP (P = .422) (Table 2) \| \| --- \| --- \|   **Is the review team’s aim for this result…?**   \| □ \| **to assess the effect of *assignment to intervention* (the ‘intention-to-treat’ effect) (x)** \| \| --- \| --- \| \| □ \| to assess the effect of *adhering to intervention* (the ‘per-protocol’ effect) \|   **If the aim is to assess the effect of *adhering to intervention***, select the deviations from intended intervention that should be addressed (at least one must be checked):  □ occurrence of non-protocol interventions  □ failures in implementing the intervention that could have affected the outcome  □ non-adherence to their assigned intervention by trial participants  **Which of the following sources were obtained to help inform the risk-of-bias assessment? (tick as many as apply)**  □ **Journal article(s) with results of the trial (x)**  □ Trial protocol  □ Statistical analysis plan (SAP)  □ Non-commercial trial registry record (e.g. ClinicalTrials.gov record)  □ Company-owned trial registry record (e.g. GSK Clinical Study Register record)  □ “Grey literature” (e.g. unpublished thesis)  □ Conference abstract(s) about the trial  □ Regulatory document (e.g. Clinical Study Report, Drug Approval Package)  □ Research ethics application  □ Grant database summary (e.g. NIH RePORTER or Research Councils UK Gateway to Research)  □ Personal communication with trialist  □ Personal communication with the sponsor |
| --- | --- | --- | --- | --- | --- | --- | --- | --- | --- | --- | --- | --- | --- | --- | --- | --- | --- | --- | --- | --- |

Risk of bias assessment

Responses underlined in green are potential markers for low risk of bias, and responses in red are potential markers for a risk of bias. Where questions relate only to sign posts to other questions, no formatting is used.

**Domain 1: Risk of bias arising from the randomization process**

| **Signalling questions** | **Comments** | **Response options** |
| --- | --- | --- |
| **1.1 Was the allocation sequence random?** | The study explicitly states that participants were "simply randomized into three matched groups according to the applied therapeutic modality using a computer-generated list." This indicates a truly random sequence.  The study reports that "concealment of allocation was safeguarded by enveloping assignments in sequentially numbered, opaque sealed envelopes, which were only opened once the study was settled." This is standard allocation concealment. | Y |
| **1.2 Was the allocation sequence concealed until participants were enrolled and assigned to interventions?** |  | Y |
| **1.3 Did baseline differences between intervention groups suggest a problem with the randomization process?** | Table 1 shows that the three groups were well matched regarding gender, age, duration, keloid site, etiology, family history, and surface area (all P > 0.05), suggesting successful randomization without baseline imbalance. | N |
| **Risk-of-bias judgement** | - Random sequence generation and allocation concealment were explicitly described and appropriate. - Baseline characteristics were similar across groups. - No issues suggest the randomization process introduced bias. | Low |
| Optional: What is the predicted direction of bias arising from the randomization process? |  | NA |

Domain 2: Risk of bias due to deviations from the intended interventions (*effect of assignment to intervention*)

| **Signalling questions** | **Comments** | **Response options** |
| --- | --- | --- |
| **2.1. Were participants aware of their assigned intervention during the trial?** |  | Y / PY / PN / N / NI |
| **2.2. Were carers and people delivering the interventions aware of participants' assigned intervention during the trial?** |  | Y / PY / PN / N / NI |
| **2.3. If Y/PY/NI to 2.1 or 2.2: Were there deviations from the intended intervention that arose because of the trial context?** |  | NA / Y / PY / PN / N / NI |
| **2.4 If Y/PY to 2.3: Were these deviations likely to have affected the outcome?** |  | NA / Y / PY / PN / N / NI |
| **2.5. If Y/PY/NI to 2.4: Were these deviations from intended intervention balanced between groups?** |  | NA / Y / PY / PN / N / NI |
| **2.6 Was an appropriate analysis used to estimate the effect of assignment to intervention?** |  | Y / PY / PN / N / NI |
| **2.7 If N/PN/NI to 2.6: Was there potential for a substantial impact (on the result) of the failure to analyse participants in the group to which they were randomized?** |  | NA / Y / PY / PN / N / NI |
| **Risk-of-bias judgement** |  | Low / High / Some concerns |
| Optional: What is the predicted direction of bias due to deviations from intended interventions? |  | NA / Favours experimental / Favours comparator / Towards null /Away from null / Unpredictable |

Domain 2: Risk of bias due to deviations from the intended interventions (*effect of adhering to intervention*)

| **Signalling questions** | **Comments** | **Response options** |
| --- | --- | --- |
| **2.1. Were participants aware of their assigned intervention during the trial?** | The paper states: *“It was unsuitable to blind the patients about the selected treatment regimen because blood sample collection was requested in PRP group only.”*  There is no mention of blinding the clinicians delivering the injections. The preparation and administration of PRP, BTX-A, and TAC differ, and so it is highly likely that the treating dermatologists **were aware**. | Y |
| **2.2. Were carers and people delivering the interventions aware of participants' assigned intervention during the trial?** |  | Y |
| **2.3. [If applicable:] If Y/PY/NI to 2.1 or 2.2: Were important non-protocol interventions balanced across intervention groups?** | The paper doesn’t report any deviations caused by participants’ or clinicians’ awareness. The interventions were applied as planned. | N |
| **2.4. [If applicable:] Were there failures in implementing the intervention that could have affected the outcome?** | Not applicable because 2.3 = N. | NA |
| **2.5. [If applicable:] Was there non-adherence to the assigned intervention regimen that could have affected participants’ outcomes?** | Not applicable because 2.3 = N. | NA |
| **2.6. If N/PN/NI to 2.3, or Y/PY/NI to 2.4 or 2.5: Was an appropriate analysis used to estimate the effect of adhering to the intervention?** | The study used **per-protocol analysis**, comparing only patients who completed the study; intention-to-treat (ITT) analysis was **not mentioned**. In RoB 2.0, the “effect of assignment” (intention-to-treat effect) requires ITT analysis. | NA |
| **Risk-of-bias judgement** |  | Some concerns |
| Optional: What is the predicted direction of bias due to deviations from intended interventions? | Since there were no deviations affecting outcomes, the bias is unlikely to favor any group. | NA |

Domain 3: Missing outcome data

| **Signalling questions** | **Comments** | **Response options** |
| --- | --- | --- |
| **3.1 Were data for this outcome available for all, or nearly all, participants randomized?** | The study enrolled **60 patients** divided into three groups of 20 each. There is no mention of any participant dropout or missing data for the primary outcomes (VSS, VRS, dermoscopic, histopathology, CTGF expression). | Y |
| **3.2 If N/PN/NI to 3.1: Is there evidence that the result was not biased by missing outcome data?** |  | NA |
| **3.3 If N/PN to 3.2: Could missingness in the outcome depend on its true value?** |  | NA |
| **3.4 If Y/PY/NI to 3.3: Is it likely that missingness in the outcome depended on its true value?** |  | NA |
| **Risk-of-bias judgement** |  | Low |
| Optional: What is the predicted direction of bias due to missing outcome data? |  | NA |

Domain 4: Risk of bias in measurement of the outcome

| **Signalling questions** | **Comments** | **Response options** |
| --- | --- | --- |
| **4.1 Was the method of measuring the outcome inappropriate?** | **Outcome measurement methods:** Vancouver Scar Scale (VSS), Verbal Rating Scale (VRS), dermoscopic examination, histopathology, and immunohistochemistry of CTGF.  **Comment:** These are standard, validated measures for assessing keloid severity, clinical improvement, and histological changes. | N |
| **4.2 Could measurement or ascertainment of the outcome have differed between intervention groups?** | **Comment:** Clinical assessment was performed by **two independent blinded dermatologists**, which minimizes differences between groups. Histopathological and immunohistochemical analysis is objective. | N |
| **4.3 If N/PN/NI to 4.1 and 4.2: Were outcome assessors aware of the intervention received by study participants?** | **Comment:** The study is **single-blind**. The dermatologists assessing clinical outcomes were blinded. However, patients were **not blinded** (especially PRP group due to blood collection). Histopathology and immunohistochemistry were presumably blinded to intervention, though not explicitly stated. | N |
| **4.4 If Y/PY/NI to 4.3: Could assessment of the outcome have been influenced by knowledge of intervention received?** | **Comment:** Since outcome assessors were blinded, influence of knowledge is unlikely. | NA |
| **4.5 If Y/PY/NI to 4.4: Is it likely that assessment of the outcome was influenced by knowledge of intervention received?** |  | NA |
| **Risk-of-bias judgement** |  | Low |
| Optional: What is the predicted direction of bias in measurement of the outcome? |  | NA |

Domain 5: Risk of bias in selection of the reported result

| **Signalling questions** | **Comments** | **Response options** |
| --- | --- | --- |
| **5.1 Were the data that produced this result analysed in accordance with a pre-specified analysis plan that was finalized before unblinded outcome data were available for analysis?** | **Comment:** The paper does not mention any pre-registered protocol, pre-specified statistical analysis plan, or trial registration. Therefore, it is unclear whether the analyses were planned in advance or could have been influenced by observed results. | NI |
| **Is the numerical result being assessed likely to have been selected, on the basis of the results, from...** |  |  |
| **5.2. ... multiple eligible outcome measurements (e.g. scales, definitions, time points) within the outcome domain?** | The primary outcomes seem to be **Vancouver Scar Scale (VSS)**, **Verbal Rating Scale (VRS)**, and CTGF immunohistochemistry. These are standard measures for keloid treatment studies. Although multiple components exist (e.g., vascularity, pigmentation, pliability, height in VSS), the paper reports them comprehensively, without evidence that only favorable outcomes were selectively reported. | PN |
| **5.3 ... multiple eligible analyses of the data?** | The analyses seem straightforward: comparisons between groups were performed using appropriate statistical tests (t-test, chi-square, Mann-Whitney, Spearman). There is no evidence that multiple post-hoc analyses were performed and only favorable ones reported. Subgroup analyses were limited to clinically relevant correlations. | PN |
| **Risk-of-bias judgement** | The study does not report a pre-specified analysis plan or trial registration. Although the outcomes reported appear standard and the analyses are plausible, the lack of a pre-specified plan introduces some risk that results could have been selectively reported. | Some concerns |
| Optional: What is the predicted direction of bias due to selection of the reported result? | - It is unclear if selective reporting would favor BTX-A, PRP, or TAC. All outcomes reported show benefit for BTX-A and PRP, but without access to unreported outcomes or protocol, the direction of potential bias cannot be predicted confidently. | Unpredictable |

Overall risk of bias

| **Risk-of-bias judgement** | - **Domains 1, 3, and 4 (Randomization, Missing data, Measurement of outcome):** Low risk. - **Domain 2 (Deviations from intended interventions):** Some concerns due to lack of patient blinding (especially PRP group) and per-protocol analysis instead of intention-to-treat. - **Domain 5 (Selection of reported result):** Some concerns because no pre-specified analysis plan or trial registration was reported, leaving a possibility of selective reporting. | Some concerns |
| --- | --- | --- |
| Optional: What is the overall predicted direction of bias for this outcome? | While all reported results favor BTX-A and PRP over TAC, the absence of a pre-specified analysis plan means it is unclear whether any unreported outcomes or analyses could have changed the interpretation. | Unpredictable |


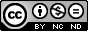


This work is licensed under a [Creative Commons Attribution-NonCommercial-NoDerivatives 4.0 International License](http://creativecommons.org/licenses/by-nc-nd/4.0/).

# (ROBINS-I V2) El-Orabi, et al., 2022

The Risk Of Bias In Non-randomized Studies – of Interventions, Version 2 (ROBINS-I V2) assessment tool

(for follow-up studies)

**20 November 2025**


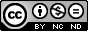


This work is licensed under a [Creative Commons Attribution-NonCommercial-NoDerivatives 4.0 International License](http://creativecommons.org/licenses/by-nc-nd/4.0/).

The ROBINS-I V2 tool

| **Reviewer:** Alessandro Alvarado AND Virgilio Blandon |
| --- |
| **Date:** January 6th of 2026 |
| **Author / Year:** Riwina Adel El-Orabi, Mohamed Radwan El-Hadidy, Ahmed Hassan El-Sabbagh., 2022  **Title:** Assessment and management of immature facial scars by  non-surgical methods |
| **Record Number:** [Internal ID] |

At planning stage: list confounding factors

P1. List the important confounding factors relevant to all or most studies on this topic. Specify whether these are particular to specific intervention-outcome combinations.

| **Important confounding factors relevant to all or most studies on this topic:**   1. **Baseline scar severity and characteristics:** This is a fundamental confounder applicable to all interventions and outcomes. More severe, hypertrophic, or larger scars at baseline have a different healing potential and response to any therapy compared to mild, flat, or small scars. 2. **Scar location on the face:** Healing and cosmetic outcomes vary significantly by facial subunit (e.g., cheek vs. perioral area vs. forehead) due to differences in skin tension, mobility, and vascularity. This affects all interventions. 3. **Mechanism of initial injury:** The nature of the trauma (e.g., laceration, burn, abrasion) determines the depth of injury, degree of inflammation, and baseline healing trajectory, influencing outcomes regardless of the intervention. 4. **Time since epithelialization (scar age) at treatment initiation:** The biological maturity of the scar is a continuum. A scar treated at 1 month post-injury is fundamentally different from one treated at 5 months, affecting plasticity and response to any anti-fibrotic or regenerative therapy. This is critical for all interventions targeting the "immature" phase. 5. **Patient age:** Age significantly influences wound healing biology, collagen remodeling, and skin elasticity. Younger patients may have a more robust healing response, potentially confounding outcomes across all interventions. 6. **Patient skin phototype (Fitzpatrick scale):** Skin type influences pigmentation changes (dyschromia), risk of post-inflammatory hyperpigmentation, and potentially collagen response. This is a key confounder for interventions affecting pigmentation (e.g., lasers) but also relevant for overall cosmetic outcomes. 7. **Genetic predisposition to abnormal scarring:** A personal or family history of hypertrophic scarring or keloids is a major determinant of scar outcome, independent of the treatment received. This is a critical confounder for all interventions. 8. **Concomitant therapies or behaviors:** The use of other scar management modalities (e.g., sunscreen, massage, silicone sheets) or health behaviors (e.g., smoking, nutrition) during the study period can significantly influence the outcome and confound the effect of the index intervention. 9. **Clinician/operator skill and technique:** For operator-dependent interventions like intralesional injections (PRP, corticosteroids) or laser therapy, the skill, experience, and technique of the practitioner are major sources of variability in outcome. This is a key confounder for specific interventions but can affect all. 10. **Intervention protocol variability (specific to certain interventions):** For biologics like PRP, factors such as preparation method (e.g., centrifugation protocol, activator use), concentration of platelets/growth factors, injection volume, depth, and number/frequency of sessions are not standardized. This variability is a massive confounder when comparing studies or groups using PRP, but less so for standardized topical agents like silicone gel. |
| --- |

For each study result: preliminary considerations (parts A to D)

**A. Specify the result being assessed for risk of bias**

A1. Specify the numerical result being assessed

| The primary comparative result is the set of p-values reported in Table 4 of the manuscript, indicating no statistically significant difference in Patient and Observer Scar Assessment Scale (POSAS) scores between the non-randomized treatment groups at the 6-month follow-up. Specifically:  **Total POSAS Score (Observer + Patient):** p = 0.58 (Kruskal-Wallis test).  **Observer Scar Scale Subscore (Total 1):** p = 0.64.  **Patient Scar Scale Subscore (Total 2):** p = 0.48.  **Observer Rating:** p = 0.46.  **Patient Satisfaction:** p = 0.58.  These p-values > 0.05 support the authors' conclusion that "The method used to treat scars showed no significant difference concerning scar evaluation." This comparative claim is the focus of the bias assessment. |
| --- |

A2. Provide further details about this result (for example, location in the study report, reason it was chosen) [optional]

| This result is located in **Table 4** of the results section, explicitly under the column "Kruskal-Wallis test P," and is described narratively in the accompanying text: "The method used to treat scars showed no significant difference concerning scar evaluation. (Table 4)." It was chosen for assessment because it represents the study's primary comparative conclusion regarding the equivalence or non-inferiority of different non-surgical interventions for immature facial scars, which is a central claim of the research. |
| --- |

A3. Specify the outcome to which this result relates

| The result relates to the comparative assessment of **scar quality** at the **6-month follow-up**, as measured by the **Patient and Observer Scar Assessment Scale (POSAS)**.  Specifically, the outcome is the **total score and component subscores** of the POSAS, which include:  **Total POSAS Score:** The sum of the Observer Scar Scale total (Total 1) and the Patient Scar Scale total (Total 2).  **Observer Scar Scale Subscore (Total 1):** The sum of ratings for vascularity, pigmentation, thickness, pliability, and relief.  **Patient Scar Scale Subscore (Total 2):** The sum of ratings for pain, itching, color, stiffness, irregularity, and thickness.  **Overall Observer Rating:** A single global rating of the scar by the observer.  **Overall Patient Satisfaction:** A single global rating of satisfaction by the patient.  The reported p-values indicate that no statistically significant differences were found between the different non-surgical treatment groups (silicone gel, corticosteroid injection, laser, and combined therapies) for any of these POSAS outcome measures at the 6-month time point. |
| --- |

**B. Decide whether to proceed with a risk-of-bias assessment**

| **Question** | **Comments** | **Response options** |
| --- | --- | --- |
| **B1 Did the authors make any attempt to control for confounding in the result being assessed?** | The study is a non-randomized clinical series where treatment was assigned based on clinician judgment and scar characteristics (e.g., "strict selection of treatment methods according to the scar characteristics"). The authors state that due to this selection, statistical measures were "insignificant between patients." However, this is a description of the study design (treatment allocation by indication) rather than an active attempt to *control for* confounding in the analysis. The statistical test used (Kruskal-Wallis) simply compares the outcome scores across the different treatment groups; it does not adjust for any baseline differences or potential confounding variables between those groups. No methods like restriction, matching, stratification, or multivariable regression were employed to address the confounders listed in the planning stage. | PN |
| **B2 If N/PN to B1: Is there sufficient potential for confounding that this result should not be considered further?** | **Comments:** There is **very high potential for confounding** that critically undermines the validity of the comparative result (that treatments show "no significant difference"). The treatment groups were almost certainly not comparable at baseline. Key confounders like **baseline scar severity, scar location, mechanism of injury, scar age, and genetic predisposition** directly influenced which treatment a patient received (e.g., corticosteroids for keloids, silicone for recent hypertrophic scars, laser/PRP for diffuse scars). This "confounding by indication" means that the groups being compared started with fundamentally different prognoses. Any similarity in final POSAS scores is far more likely explained by these pre-existing differences than by a true equivalence of the treatments. Therefore, the result is not a reliable estimate of the comparative effect of the interventions. | Y |
| **B3 Was the method of measuring the outcome inappropriate?** | **Comments:** The Patient and Observer Scar Assessment Scale (POSAS) is a validated, patient-reported outcome measure specifically designed for scar assessment. It is an appropriate tool for measuring the outcome of interest (scar quality). A minor concern is that the observer component was completed by paramedical staff with no prior experience, though they were allowed to read the instructions, which may introduce some measurement variability. However, the tool itself is not inappropriate. | PN |

**If the answer to either B2 or B3 is ‘Yes’ or ‘Probably yes’, the result should be considered to be at ‘Critical risk of bias’ and no further assessment is required.**

**C. Specify the (hypothetical) target randomized trial specific to the study**

The target randomized trial is either explicitly described by the primary study investigators or implied by the study’s design and analysis. Alongside specifying the target trial,

| C1. Specify the participants and eligibility criteria | A randomized controlled trial, ideally using an intra-patient (split-scar) design, to evaluate the effectiveness of individual non-surgical modalities compared to natural evolution (control) for improving the quality of post-traumatic immature facial scars.  **Eligibility criteria**  **Participants:** Patients presenting with at least **two comparable post-traumatic immature facial scars** (e.g., in a similar anatomical location, with equivalent injury mechanism, baseline severity, and age since epithelialization).  **Inclusion Criteria:** Individuals of any age or sex with linear or small facial scars less than 6 months old, resulting from trauma (e.g., laceration, abrasion). Both scars in the same patient must be eligible for the same type of intervention.  **Exclusion Criteria:** Patients with known scarring disorders (keloids), full-thickness burn scars, unrealistic expectations, mental disorders, or an inability to follow the protocol. |
| --- | --- |

| C2. Specify the intervention strategy | For each patient, one of the two comparable scars would be randomly assigned to receive one of the following standardized active interventions, applied as monotherapy:  **Topical silicone gel application:** Twice daily with massage, initiated shortly after randomization.  **Intralesional corticosteroid injection:** Triamcinolone acetonide 40 mg/mL, with a defined protocol for the number and timing of sessions.  **Fractional CO2 laser (FCL) therapy:** With a standardized protocol for energy, density, and number of sessions.  **Intralesional Platelet-Rich Plasma (PRP) injection:** Prepared and administered according to a standardized protocol. |
| --- | --- |
| C3. Specify the comparator strategy | The **other comparable scar** in the same patient would serve as the control. This scar would receive a protocol of **"observation and standard care"** (e.g., gentle washing, basic moisturization, and sun protection), **specifically excluding any active, scar-improving interventions** under evaluation (i.e., none of the treatments listed in C2). This design optimally controls for all patient-level confounders (age, genetics, phototype, behavior). |

C4. Did the analysis account for switches during follow-up between the intervention strategies being compared, or for other protocol deviations during follow-up?

| □ | No (the analysis is estimating the intention-to-treat effect) |
| --- | --- |
| **□** | **Yes (the analysis is estimating the per-protocol effect) (x)** |

**D. Information sources**

Which of the following sources have you obtained to help you inform your risk of bias judgements (tick as many as apply)?

- **Journal article(s) (x)**
- Study protocol
- Statistical analysis plan (SAP)
- Non-commercial registry record (e.g. ClinicalTrials.gov record)
- Company-owned registry record (e.g. GSK Clinical Study Register record)
- “Grey literature” (e.g. unpublished thesis)
- Conference abstract(s)
- Regulatory document (e.g. Clinical Study Report, Drug Approval Package)
- Individual participant data
- Research ethics application
- Grant database summary (e.g. NIH RePORTER, Research Councils UK Gateway to Research)
- Personal communication with investigator
- Personal communication with sponsor

Please specify any additional sources not listed above

|  |
| --- |

**Risk-of-bias assessment**

**Evaluation of confounding factors**

Complete a row for each important confounding factor listed in advance (subsection (i) below); and either relevant to the setting of this particular study or identified by the study authors (subsection (ii)). **“Important” confounding factors are those for which, in the context of this study,** **adjustment is expected to lead to a meaningful change in the estimated effect of the intervention.**

| **(i) Important confounding factors listed in advance** | | | | | | |
| --- | --- | --- | --- | --- | --- | --- |
| Confounding factor | Measured variable(s) for this factor, if any | Was this variable (or were these variables) controlled for in the analysis?  (Y / N) | If this confounding factor was controlled for, was it measured validly and reliably by this variable (or these variables)?*  (NA / Y / PY / PN / N / NI) | If this confounding factor was not controlled for, is there evidence that controlling for it was unnecessary?**  (NA / Y / PY / PN / N) | OPTIONAL: Is failure to adjust for this confounding factor expected to bias the effect estimate upwards or downwards? (Upward bias (overestimate the intervention effect) / Downward bias (underestimate the intervention effect) / No information or unpredictable) | Comments |
| Baseline scar severity and characteristics | Scar type/characteristics (implied by clinical description). No standardized scale reported. | N | NA | NA | Unpredictable | Treatment was selected *based on* these characteristics (e.g., corticosteroids for keloids, silicone for recent hypertrophic scars). This is the core of confounding by indication. No statistical control was applied. |
| Scar location on the face | Facial scar (inclusion criterion). Specific subunit (e.g., cheek, forehead) not detailed in analysis. | N | NA | PN | Unpredictable | While all scars were facial, outcomes vary by subunit. Location likely influenced treatment choice (e.g., ease of laser vs. silicone application) and outcome, but its distribution across groups and association with outcome is not analyzed. |
| Mechanism of initial injury | Trauma mechanism (collected in history). | N | NA | N | Unpredictable | Mechanism (e.g., burn vs. laceration) determines healing trajectory. It was not used in the analysis to adjust comparisons between treatment groups. |
| Time since epithelialization (scar age) at treatment initiation | Scar duration <6 months (inclusion). Exact timing not used analytically. | N | NA | N | Unpredictable | A key confounder. Treatment was initiated at different times within the 6-month window based on presentation, and this "scar maturity" directly affects outcome. Not controlled for. |
| Patient age | Age in years (reported as mean). | N | NA | PN | Unpredictable | Age was reported but not included as a covariate in the comparative analysis (Kruskal-Wallis test compares groups without adjustment). |
| Patient skin phototype (Fitzpatrick scale) | Not mentioned in the study. | N | N | N | Unpredictable | A critical factor for pigmentation outcomes, especially after laser. Not measured, therefore not controlled. Its absence is a significant source of potential bias. |
| Genetic predisposition to abnormal scarring | Personal/family history of keloids/hypertrophic scars - not mentioned. | N | N | N | Unpredictable | A major determinant of scar outcome. Not measured or reported, so it cannot be controlled for. |
| Concomitant therapies or behaviors | Use of other scar modalities (e.g., sunscreen, massage) not reported or controlled. | N | NA | N | Unpredictable | These behaviors (e.g., sun exposure) significantly influence scar pigmentation and maturation. No mention of standardization or measurement, so they remain uncontrolled confounders. |
| Clinician/operator skill and technique | Implied to be within a single center. Not measured or analyzed. | N | NA | PN | Unpredictable | While a single-center design reduces variability, operator skill is a known major confounder for operator-dependent interventions like injections and lasers. It was not addressed in the design or analysis. |
| Intervention protocol variability (e.g., PRP preparation) | Described in methods but variability between patients not accounted for. | N | NA | N | Unpredictable | Especially critical for PRP and laser settings. The analysis treats each "intervention group" as homogeneous, while in practice, dose, concentration, and technique varied. This is an uncorrected source of measurement error and confounding. |

* “Validity” refers to whether the confounding variable or variables accurately measure the confounding factor, while “reliability” refers to the precision of the measurement (more measurement error means less reliability).

** In the context of a particular study, variables need not be included in the analysis: (a) ) if they are measured validly and reliably and are not associated with the outcome, conditional on intervention (noting that lack of a statistically significant association is not evidence of a lack of association; (b) if they are measured validly and reliably and are not associated with intervention; (c) if they are measured validly and reliably and adjustment makes no or minimal difference to the estimated effect of the primary parameter; (d) because the confounder was addressed in the study design, for example by restricting to individuals with the same value of the confounder; (e) because a negative control demonstrates that there was unlikely to have been confounding due to this variable or that uncontrolled confounding was likely to be minimal; or (f) because external evidence suggests that controlling for the variable is not necessary in the context of the study being assessed”.

| **(ii) Additional important confounding factors relevant to the setting of this particular study, or identified by the study authors** | | | | | | |
| --- | --- | --- | --- | --- | --- | --- |
| Confounding factor | Measured variable(s) for this factor, if any | Was this variable (or were these variables) controlled for in the analysis?  (Y / N) | If this confounding factor was controlled for, was it measured validly and reliably by this variable (or these variables)?*  (NA / Y / PY / PN / N / NI) | If this confounding factor was not controlled for, is there evidence that controlling for it was unnecessary?**  (NA / Y / PY / PN / N) | OPTIONAL: Is failure to adjust for this confounding factor expected to bias the effect estimate upwards or downwards? (Upward bias (overestimate the intervention effect) / Downward bias (underestimate the intervention effect) / No information or unpredictable) | Comments |
| Patient Sex | Sex (male/female) - reported in demographics. | N | NA | PN | Unpredictable | The authors discuss sex differences in healing in the discussion, implying it's a relevant factor. It was reported but not included as a covariate in the comparative analysis between treatment groups. |

* “Validity” refers to whether the confounding variable or variables accurately measure the confounding factor, while “reliability” refers to the precision of the measurement (more measurement error means less reliability).

** In the context of a particular study, variables need not be included in the analysis: (a) ) if they are measured validly and reliably and are not associated with the outcome, conditional on intervention (noting that lack of a statistically significant association is not evidence of a lack of association; (b) if they are measured validly and reliably and are not associated with intervention; (c) if they are measured validly and reliably and adjustment makes no or minimal difference to the estimated effect of the primary parameter; (d) because the confounder was addressed in the study design, for example by restricting to individuals with the same value of the confounder; (e) because a negative control demonstrates that there was unlikely to have been confounding due to this variable or that uncontrolled confounding was likely to be minimal; or (f) because external evidence suggests that controlling for the variable is not necessary in the context of the study being assessed”.

**Signalling questions and risk-of-bias judgements**

Responses underlined in green are potential markers for low risk of bias, and responses in red are potential markers for a risk of bias. Where questions relate only to sign posts to other questions, no formatting is used.

**1. Bias due to confounding**

**Domain 1, Variant A (the analysis is estimating the intention-to-treat effect so only baseline confounding needs to be addressed – if No to C4)**

| **Signalling questions** | **Comments** | **Response** |
| --- | --- | --- |
| **1.1 Did the authors control for all the important confounding factors for which this was necessary?** | The analysis did not control for any of the pre-specified or additional important confounding factors. Treatment was assigned based on clinical judgment and scar characteristics ("strict selection...according to the scar characteristics"), creating classic confounding by indication. Critical factors like baseline scar severity, location, mechanism, scar age, skin phototype, and genetic predisposition were not adjusted for. The authors' explanation that results were insignificant *due to* this selection process confirms that these confounders were strongly associated with both the intervention and the outcome, making uncontrolled confounding substantial. | SN (no, and uncontrolled confounding was probably substantial) |
| **1.2 If Y/PY/WN to 1.1: Were confounding factors that were controlled for (and for which control was necessary) measured validly and reliably by the variables available in this study?** | This question is not applicable because the answer to 1.1 is "SN" (No, and uncontrolled confounding was probably substantial). No confounders were controlled for in the analysis. | NA |
| **1.3 If Y/PY/WN to 1.1: Did the authors control for any post-intervention variables that could have been affected by the intervention?** | This question is not applicable because the answer to 1.1 is "SN". The analysis did not control for any variables, whether pre- or post-intervention. | NA |
| **1.4. Did the use of negative controls, quantitative bias analysis, or other considerations, suggest serious uncontrolled confounding?** | The study design itself acts as a strong indicator. The explicit treatment assignment strategy—where specific interventions (e.g., corticosteroids, laser+PRP) were reserved for more severe, hypertrophic, or complex scars—constitutes a powerful, built-in negative control. This practice ensures that prognostic factors are inextricably linked to treatment choice. The authors' own narrative confirms this link. Therefore, other considerations (the study's design and stated methods) strongly suggest serious uncontrolled confounding. | Y |
| Risk of bias judgement |  | Critical |
| Optional: What is the predicted direction of bias due to confounding? | The direction is complex and likely differs for each treatment comparison. For instance, corticosteroids were used for the worst scars (keloids/hypertrophic), which could bias its effect *downward* (making it seem less effective) compared to silicone gel used for milder scars. Conversely, comparing a single-modality group to a combination therapy group (which likely received treatment for more complex scars) could bias the combination's effect downward. With multiple confounders (severity, location, age, phototype) pulling in different directions across multiple treatment comparisons, the net direction of bias for the overall conclusion of "no difference" is not predictable with confidence. | Unpredictable |

**Domain 1, Variant B (the analysis is estimating the per-protocol effect so both baseline and time-varying confounding need to be addressed – if Yes to C4)**

**Variant B of Domain 1 is used when the analysis accounted for protocol deviations such as switches during follow-up between the intervention strategies being compared, or stopping active intervention without a clinical indication to do so. Accounting for protocol deviations might be done for example by partitioning follow-up for individual participants according to the intervention received, or by censoring follow-up when participants deviated from their initial intervention. It is then necessary to control for both baseline and time—varying confounding.**

| **Signalling questions** | **Comments** | **Response** |
| --- | --- | --- |
| **1.1 Did the authors use an analysis method that was appropriate to control for time-varying as well as baseline confounding?** | The analysis method used was a simple Kruskal-Wallis test comparing outcome scores across treatment groups. This method does not adjust for any confounding variables, whether baseline or time-varying. It assumes groups are comparable at baseline, which they are not in this non-randomized study with treatment assignment based on scar characteristics. | N |
| **1.2 If Y/PY to 1.1: Did the authors control for all the important baseline and time-varying confounding factors for which this was necessary?** | This question is not applicable because the answer to 1.1 is "N". | NA |
| **1.3 If Y/PY/WN to 1.2: Were confounding factors that were controlled for (and for which control was necessary) measured validly and reliably by the variables available in this study?** | This question is not applicable because the answer to 1.1 is "N". | NA |
| **1.4 If N/PN/NI to 1.1: Did the authors control for time-varying factors or other variables measured after the start of intervention?** | The authors did not control for any variables measured after the start of intervention. The analysis was a simple comparison of final outcomes between groups without any adjustment for factors that might have changed during follow-up. | N |
| **1.5 Did the use of negative controls, or other considerations, suggest serious uncontrolled confounding?** | The study's own design and description serve as strong evidence of serious uncontrolled confounding. The authors explicitly state that treatments were selected based on scar characteristics ("strict selection of treatment methods according to the scar characteristics"). This means that prognostic factors (scar severity, type, etc.) directly determined treatment assignment, creating fundamental confounding by indication. | Y |
| Risk of bias judgement |  | Critical |
| Optional: What is the predicted direction of bias due to confounding? | The direction of bias is complex and likely varies across different treatment comparisons. More aggressive treatments (like corticosteroids, laser+PRP) were used for more severe scars, which could bias their apparent effect downward relative to simpler treatments used for milder scars. However, with multiple confounding factors operating simultaneously across multiple treatment groups, the net direction of bias for the overall conclusion of "no significant difference" cannot be confidently predicted. | Unpredictable |

**2. Bias in classification of interventions**

| **Signalling questions** | **Comments** | **Response options** |
| --- | --- | --- |
| **2.1 Were the intervention strategies distinguishable at the time when follow-up would have started in the target trial?** | In the target trial, interventions would be assigned at baseline. In this study, treatment was assigned and initiated at the start of follow-up, and the interventions (silicone gel, corticosteroid injection, laser, PRP, and combinations) are distinct and clearly defined. | Y |
| **2.2 If N/PN/NI to 2.1: Did all or nearly all outcome events occur after the intervention and comparator strategies could be distinguished?** | Not applicable. | NA |
| **2.3 If N/PN/NI to 2.2: Did the analysis avoid problems arising from intervention strategies that are not distinguishable at the start of follow-up?** | Not applicable. | NA |
| **2.4 Was classification of intervention status influenced by knowledge of the outcome or risk of the outcome?** | Treatment assignment was based on scar characteristics (a risk factor), but the recording of the intervention received was made at the time of treatment, prior to outcome assessment. There is no indication that outcome knowledge influenced the classification of intervention status. | N |
| **2.5 Were further classification errors (not influenced by knowledge of the outcome or risk of the outcome) likely?** | The interventions are distinct, but adherence to topical treatments (e.g., silicone gel) was not monitored or reported. Non-adherence could lead to misclassification of treatment received. The possibility of such errors exists, but their extent is unclear. | PY |
| Risk of bias judgement | The intervention groups are clearly defined and recorded at baseline, and there is no evidence of differential misclassification. However, potential non-differential misclassification (e.g., due to non-adherence) could bias effect estimates toward the null. | Moderate |
| Optional: What is the predicted direction of bias in classification of interventions? | Any non-differential misclassification would likely bias the estimated differences between treatment groups toward the null (i.e., toward finding no difference). | Towards null |

Algorithm for reaching default risk of bias judgement:

**3. Bias in selection of participants into the study (or into the analysis)**

| **Signalling questions** | **Comments** | **Response options** |
| --- | --- | --- |
| *A. Questions about prevalent user bias and immortal time* |  |  |
| **3.1 Did follow up in the analysis begin at the start of the intervention strategies being compared?** | Follow-up for outcome assessment (POSAS) began at the start of each patient's intervention. The outcome was measured at 6 months post-treatment. While the exact timing of intervention start relative to injury varied, the analysis effectively treats the start of intervention as time zero for each patient. There is no indication of immortal time bias. | Y |
| **3.2 If Y/PY to 3.1:** **Were outcome events during a period of follow-up after the start of the interventions excluded from the analysis?** | The outcome is a single assessment at 6 months. There is no continuous monitoring of "events" over time, so no outcome events were excluded. | N |
| *B. Questions about other types of selection bias* |  |  |
| **3.3 Was selection of participants into the study (or into the analysis) based on participant characteristics observed after the start of intervention (additional to the situations addressed in 3.1 and 3.2)?** | Participants were included based on baseline criteria (facial scar <6 months). Treatment assignment was based on baseline scar characteristics, but this is a confounding issue (Domain 1). No selection into the study or analysis was based on post-intervention characteristics. | N |
| **3.4 If Y/PY to 3.3: Were the post-intervention variables that influenced selection likely to be associated with intervention?** | Not applicable. | NA |
| **3.5 If Y/PY to 3.4: Were the post-intervention variables that influenced selection likely to be influenced by the outcome or a cause of the outcome?** | Not applicable. | NA |
| *C. Questions about analysis, sensitivity analyses and severity of the problem* |  |  |
| **3.6 If SN to 3.1 or Y/PY to 3.5: Is it likely that the analysis corrected for all of the potential selection biases identified above?** | Not applicable | NA |
| **3.7 If N/PN/NI to 3.6: Did sensitivity analyses demonstrate that the likely impact of the potential selection biases identified above was minimal?** | Not applicable. | NA |
| **3.8 If N/PN/NI to 3.7: Were potential selection biases identified above sufficiently severe that the result should not be included in a quantitative synthesis?** | Not applicable. | NA |
| Risk of bias judgement | No evidence of selection bias due to post-intervention variables or immortal time. The main biases arise from non-random treatment assignment (confounding, Domain 1). | Low |
| Optional: What is the predicted direction of bias in selection of participants into the study? |  | Favours intervention / Favours comparator / Towards null /Away from null / Unpredictable |

**4. Bias due to missing data**

| **Signalling questions** | **Comments** | **Response options** |
| --- | --- | --- |
| **4.1 Were complete data on intervention status available for all, or nearly all, participants?** | The study reports that all 15 patients received treatment. There is no indication of missing data on intervention status. | Y |
| **4.2 Were complete data on the outcome available for all, or nearly all, participants?** | The primary outcome (POSAS scores) was assessed at 6 months for all 15 included patients. No loss to follow-up is reported. | Y |
| **4.3 Were complete data on important confounding variables available for all, or nearly all, participants?** | Many important confounding factors (e.g., skin phototype, genetic predisposition) were not measured at all. Therefore, complete data for these important confounders are not available. | N |
| **4.4 If N/PN/NI to 4.1, 4.2 or 4.3: Is the result based on a complete case analysis?** | The analysis included all 15 patients for the variables that were measured (intervention, outcome). However, it did not include the unmeasured confounders. For the available data, it is a complete case analysis. | Y |
| **4.5 If Y/PY/NI to 4.4: Was exclusion from the analysis because of missing data (in intervention, confounders or the outcome) likely to be related to the true value of the outcome?** | No participants were excluded due to missing data. The absence of confounder data is due to non-measurement, not exclusion of participants with missing values. | N |
| **4.6 If Y/PY/NI to 4.5:** **Is the relationship between the outcome and missingness likely to be explained by the variables in the analysis model?** | Not applicable. | NA |
| **4.7 If N/PN to 4.4: Was the analysis based on imputing missing values?** | Not applicable. | NA |
| **4.8 If Y/PY to 4.7: Is it reasonable to assume that data were ‘missing at random’ (MAR) or ‘missing completely at random’ (MCAR)?** | Not applicable. | NA |
| **4.9 If Y/PY to 4.8: Was imputation performed appropriately?** | Not applicable. | NA |
| **4.10 If N/PN/NI to 4.7: Was an appropriate alternative method used to correct for bias due to missing data?** | Not applicable. | NA |
| **4.11 If PN/N/NI to 4.1, 4.2 or 4.3 AND (Y/PY/NI to 4.5 OR WN/SN/NI to 4.9 OR WN/SN/NI to 4.10): Is there evidence that the result was not biased by missing data?** | Not applicable. | NA |
| Risk of bias judgement | There was no loss to follow-up, and data on intervention and outcome were complete for all participants. The lack of measurement of important confounders is a problem of confounding (Domain 1), not of missing data among measured variables. | Low |
| Optional: What is the predicted direction of bias due to missing data? |  | Favours intervention / Favours comparator / Towards null /Away from null / Unpredictable |

**5. Bias in measurement of the outcome**

| **Signalling questions** | **Comments** | **Response options** |
| --- | --- | --- |
| **5.1 Could measurement or ascertainment of the outcome have differed between intervention groups?** | The same tool (POSAS) and timing (6 months) were used for all groups. However, the lack of blinding means that knowledge of the treatment could have influenced how patients self-reported and how observers rated scars. The measurement process itself was the same, but the potential for bias in how it was applied exists. | PY |
| **5.2 Were outcome assessors aware of the intervention received by study participants?** | Yes. The patient-assessors were certainly aware of their own treatment. The observer-assessors (paramedical staff) were not blinded and were likely aware of the treatment received, as they were part of the clinical team. | Y |
| **5.3 If Y/PY/NI to 5.2: Could assessment of the outcome have been influenced by knowledge of the intervention received?** | The subjective nature of scar assessment (both patient and observer components) makes it highly susceptible to influence by expectations. Patients with more intensive treatments (e.g., laser, PRP) might rate their scars more favorably due to higher expectations or perceived investment. Observers' ratings could also be influenced by preconceptions about treatment effectiveness. | SY (yes, to a large extent) |
| Risk of bias judgement | The lack of blinding of patients and outcome assessors, combined with the subjective nature of the primary outcome measure, introduces a serious risk of measurement bias. | Serious |
| Optional: What is the predicted direction of bias in measurement of outcomes? | The direction is unpredictable. It could favor more intensive or novel treatments (due to placebo or expectation effects) or, conversely, could favor simpler treatments if more severe scars (with worse prognosis) were assigned to intensive treatments. | Unpredictable |

**6. Bias in selection of the reported result**

| **Signalling questions** | **Comments** | **Response options** |
| --- | --- | --- |
| **6.1 Was the result reported in accordance with an available, pre-determined analysis plan?** | The manuscript does not mention a pre-registered or pre-specified analysis plan. The methods section states the statistical tests used but does not provide a detailed analysis plan that was finalized before data collection or analysis. | N |
| **Is the numerical result being assessed likely to have been selected, on the basis of the results, from...** |  |  |
| **6.2 ... multiple outcome *measurements* (e.g. scales, definitions, time points) within the outcome domain?** | The result includes p-values for five different outcome measurements derived from the POSAS (Total 1, Total 2, Total 1+2, Observer rating, Patient satisfaction). Selection among these multiple measurements is possible. | Y |
| **6.3 ... multiple *analyses* of the data?** | The manuscript presents the Kruskal-Wallis test as the primary comparative analysis. No other comparative analyses (e.g., ANOVA, regression) are reported. While not explicitly stated, it is possible that other analyses were considered but not reported, but there is no direct evidence. | PN |
| **6.4 ... multiple *subgroups*?** | No subgroup analyses are reported. | N |
| Risk of bias judgement | Given the lack of a pre-specified analysis plan and the presentation of results from multiple outcome measurements, there is a risk of selective reporting. The authors report that all comparisons were non-significant, which could be a comprehensive report or a selective one. Without a protocol, it is difficult to rule out selective reporting. However, the study is small and exploratory, and the main issue is the lack of a plan rather than clear evidence of selective reporting from multiple analyses. | Moderate |
| Optional: What is the predicted direction of bias in selection of the reported result? | Unpredictable. Selective reporting could favor either finding significant differences or not. In this case, the reported result is non-significant for all comparisons. It is unclear if this reflects a true null effect or a selective report. | Unpredictable |

**Overall risk of bias**

|  | **Comment** | **Response options** |
| --- | --- | --- |
| Overall risk of bias | The overall judgment is driven by the **Critical risk of bias in Domain 1 (Bias due to confounding)**. The study's non-randomized design with treatment assignment based on scar characteristics ("confounding by indication") led to severe, uncontrolled confounding by factors such as baseline scar severity, location, and age. This fundamentally invalidates the comparative conclusion that the treatments are equivalent. Additionally, **Serious risk of bias in Domain 5 (Bias in measurement of the outcome)** due to lack of blinding further compromises the result's reliability. According to the ROBINS-I algorithm, the overall risk of bias is the most severe rating across all domains. | Critical risk |
| What is the predicted direction of bias? | The direction of bias is complex and cannot be confidently predicted. In Domain 1, the confounding likely operates in different directions for different treatment comparisons (e.g., more aggressive treatments used for worse-prognosis scars could bias their effects downward). The measurement bias in Domain 5 could also pull in unpredictable directions based on patient and observer expectations. Therefore, the net effect on the overall conclusion of "no difference" is unpredictable. | Unpredictable |


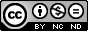


This work is licensed under a [Creative Commons Attribution-NonCommercial-NoDerivatives 4.0 International License](http://creativecommons.org/licenses/by-nc-nd/4.0/).
