# Supplementary material for: Platelet-rich plasma for immature post-traumatic scars and early keloids: A scoping review
Source: PLoS One. 2026 Apr 6;21(4):e0345754. doi: 10.1371/journal.pone.0345754 (PMC13052873; doi:10.1371/journal.pone.0345754)
Supplement: S1 Table — This table provides detailed electronic search strategies, databases, search dates, and number of records retrieved for studies evaluating platelet-rich plasma (PRP) and related platelet-derived products for early post-traumatic scar formation and wound healing. The search combined controlled vocabulary and free-text terms for PRP, scar types, tissue repair, and early phases of healing. Additional records were identified through backward and forward citation tracking. The total number of records retrieved across all sources was 1,325. (DOCX) [file pone.0345754.s004.docx]

# **S1 Table. Search Strategy Overview and Results Across Electronic Databases**

| **Dataset** | **Date** | **Search String** | **Results** |
| --- | --- | --- | --- |
| PubMed/MEDLINE | 11/11/2025 | ("platelet-rich plasma"[tiab] OR "PRP"[tiab] OR "autologous platelet concentrate"[tiab] OR "APC"[tiab] OR "platelet gel"[tiab] OR "platelet concentrate"[tiab] OR "thrombocyte concentrate"[tiab] OR "autologous conditioned plasma"[tiab] OR "platelet-derived growth factors"[tiab] OR "platelet lysate"[tiab] OR "platelet-rich fibrin"[tiab] OR "PRF"[tiab] OR "platelet-rich matrix"[tiab] OR "autologous blood concentrate"[tiab] OR "plasma rich in growth factors"[tiab]) AND ("scar"[tiab] OR "cicatrix"[tiab] OR "cicatrices"[tiab] OR "scarring"[tiab] OR "scar formation"[tiab] OR "scar tissue"[tiab] OR "fibrosis"[tiab] OR "dermal fibrosis"[tiab] OR "cutaneous scar"[tiab] OR "skin scar"[tiab] OR "post-traumatic"[tiab] OR "posttraumatic"[tiab] OR "traumatic scar"[tiab] OR "wound healing"[tiab] OR "wound repair"[tiab] OR "tissue repair"[tiab] OR "tissue regeneration"[tiab] OR "cutaneous healing"[tiab] OR "dermal repair"[tiab] OR "skin regeneration"[tiab]) AND ("early"[tiab] OR "recent"[tiab] OR "acute"[tiab] OR "subacute"[tiab] OR "immature"[tiab] OR "emerging"[tiab] OR "developing"[tiab] OR "formative"[tiab] OR "evolving"[tiab] OR "early phase"[tiab] OR "inflammatory phase"[tiab] OR "proliferative phase"[tiab] OR "remodeling phase"[tiab] OR "maturation phase"[tiab] OR "initial healing"[tiab] OR "primary healing"[tiab] OR "secondary healing"[tiab]) | 864 |
| DOAJ | 11/11/2025 | ("platelet-rich plasma" OR PRP OR "autologous platelet concentrate" OR "platelet gel" OR "platelet-rich fibrin") AND (scar OR scarring OR "wound healing" OR "tissue repair" OR fibrosis) AND (early OR recent OR acute OR "remodeling phase") | 0 |
| DOAJ | 11/11/2025 | "Immature scar and Plasma" | 1 |
| SciELO | 11/11/2025 | ("platelet-rich plasma" OR "plasma rico en plaquetas" OR "plasma rico em plaquetas" OR PRP OR "autologous platelet concentrate" OR "concentrado plaquetario autólogo" OR "concentrado plaquetário autólogo" OR "platelet gel" OR "gel de plaquetas" OR "gel plaquetário" OR "platelet-rich fibrin" OR "fibrina rica en plaquetas" OR "fibrina rica em plaquetas" OR "platelet concentrate" OR "concentrado de plaquetas" OR "concentrado plaquetário" OR "plasma rich in growth factors" OR "plasma rico en factores de crecimiento" OR "plasma rico em fatores de crescimento") AND (scar OR scars OR scarring OR cicatriz OR cicatrices OR cicatrización OR cicatrizes OR cicatrização OR "wound healing" OR "curación de heridas" OR "cicatrização de feridas" OR "tissue repair" OR "reparación tisular" OR "reparação tecidual" OR "tissue regeneration" OR "regeneración tisular" OR "regeneração tecidual" OR fibrosis OR "dermal fibrosis" OR "fibrosis dérmica" OR "fibrose dérmica" OR "cutaneous scar" OR "cicatriz cutánea" OR "cicatriz cutânea") AND (early OR temprano OR precoce OR recent OR reciente OR recente OR acute OR agudo OR "remodeling phase" OR "fase de remodelación" OR "fase de remodelação" OR "inflammatory phase" OR "fase inflamatoria" OR "fase inflamatória" OR "proliferative phase" OR "fase proliferativa" OR "initial healing" OR "curación inicial" OR "cicatrização inicial") | 17 |
| LILACS | 11/11/2025 | (mh:"plasma rico en plaquetas" OR "plasma rico em plaquetas" OR "platelet-rich plasma" OR PRP OR "concentrado plaquetario" OR "concentrado de plaquetas" OR "platelet gel" OR "gel plaquetário" OR "platelet concentrate" OR "concentrado plaquetário" OR "plasma rico en factores de crecimiento" OR "plasma rico em fatores de crescimento") AND (mh:cicatriz OR cicatriz OR cicatrización OR cicatrização OR scar OR scars OR scarring OR "scar formation" OR "scar tissue" OR fibrosis OR "dermal fibrosis" OR "fibrosis dérmica" OR "fibrose dérmica" OR "dermal repair" OR "tissue repair") | 368 |
| Google Scholar | 26/11/2025 | ("platelet-rich plasma" OR "autologous platelet-rich plasma" OR PRP) AND ("acute scar" OR "acute scarring" OR "early scar" OR "recent scar" OR "post-traumatic scar") AND (human OR patients OR "clinical trial") - wound - wounds - animal - rat - mouse - "in vitro" | 40 |
| Backward-Forward Citation Search |  |  | 35 |
| **Total** | — | — | **1325** |

This table provides detailed electronic search strategies, databases, search dates, and number of records retrieved for studies evaluating platelet-rich plasma (PRP) and related platelet-derived products for early post-traumatic scar formation and wound healing. The search combined controlled vocabulary and free-text terms for PRP, scar types, tissue repair, and early phases of healing. Additional records were identified through backward and forward citation tracking. The total number of records retrieved across all sources was 1,325.
