# Supplementary material for: Platelet-rich plasma for immature post-traumatic scars and early keloids: A scoping review
Source: PLoS One. 2026 Apr 6;21(4):e0345754. doi: 10.1371/journal.pone.0345754 (PMC13052873; doi:10.1371/journal.pone.0345754)
Supplement: S3 Table — This table summarizes the outcomes of the full-text screening phase. It reports the number of articles excluded after full-text assessment along with the reasons for exclusion, based on predefined eligibility criteria, including population outside the predefined timeframe (>6 months), study design, intervention characteristics, and availability of full text. Articles meeting all inclusion criteria were included for final qualitative synthesis. The table provides a quantitative overview of inclusion and exclusion decisions at the full-text screening stage. (DOCX) [file pone.0345754.s006.docx]

# **S3 Table. Full-text review inclusion and exclusion**

| **Reason for Exclusion / Status** | **Number of Articles** |
| --- | --- |
| Wrong population (>6 months) | 28 |
| Review/editorial | 0 |
| Conference abstract | 0 |
| Non-peer-reviewed | 0 |
| Animal study | 0 |
| No PRP intervention | 0 |
| Non-autologous (APC/PRP/other) | 0 |
| Insufficient data | 0 |
| Pediatrics only | 0 |
| Duplicate | 0 |
| No full text | 0 |
| Wrong study design | 0 |
| **Subtotal of Excluded** | 28 |
| **Included for final review** | 5 |
| **Total full-text articles assessed** | 33 |

This table summarizes the outcomes of the full-text screening phase. It reports the number of articles excluded after full-text assessment along with the reasons for exclusion, based on predefined eligibility criteria, including population outside the predefined timeframe (>6 months), study design, intervention characteristics, and availability of full text. Articles meeting all inclusion criteria were included for final qualitative synthesis. The table provides a quantitative overview of inclusion and exclusion decisions at the full-text screening stage.
