# Supplementary material for: Platelet-rich plasma for immature post-traumatic scars and early keloids: A scoping review
Source: PLoS One. 2026 Apr 6;21(4):e0345754. doi: 10.1371/journal.pone.0345754 (PMC13052873; doi:10.1371/journal.pone.0345754)
Supplement: S4 Table — This table reports the results of backward and forward citation searches performed on selected source articles to identify additional studies potentially eligible for inclusion. For each round, the table provides the source article ID, date of the search (forward searches only), search type (backward or forward), total results retrieved, number of new articles selected for detailed review, number of articles meeting inclusion criteria, and reasons for exclusion when applicable. N/A indicates that no comment was provided or that the information was not applicable. Exclusion reasons include wrong population (>6 months), duplicate, review/editorial, no PRP intervention, non-autologous intervention, no full text, or other study-specific criteria. (DOCX) [file pone.0345754.s007.docx]

# **S4 Table. Summary of backward and forward citation searches across databases**

| **Round** | **Date (forward only)** | **Source Article (ID)** | **Search Type** | **Total Results** | **New Articles for Detailed Review** | **Meet Inclusion Criteria** | **Exclusion Justification** | **Comments** |
| --- | --- | --- | --- | --- | --- | --- | --- | --- |
| 1 | N/A | PubMed-402 | Backward | 32 | 4 | 0 | Wrong population (>6 months, n=2); Duplicate (n=2) | N/A |
| 2 | N/A | DOAJ-1 | Backward | 27 | 0 | 0 | N/A | N/A |
| 3 | N/A | Google-1 | Backward | 26 | 0 | 0 | N/A | N/A |
| 4 | 26 Nov 2025 | PubMed-402 | Forward | 19 | 1 | 0 | Wrong population (>6 months, n=1) | N/A |
| 5 | 26 Nov 2025 | DOAJ-1 | Forward | 6 | 0 | 0 | N/A | N/A |
| 6 | 26 Nov 2025 | Google-1 | Forward | 6 | 0 | 0 | N/A | N/A |
| 7 | N/A | PubMed-1 | Backward | 202 | 1 | 0 | Wrong population (>6 months, n=1) | N/A |
| 8 | N/A | PubMed-2 | Backward | 39 | 0 | 0 | N/A | N/A |
| 9 | N/A | PubMed-4 | Backward | 342 | 0 | 0 | N/A | N/A |
| 10 | N/A | PubMed-5 | Backward | 177 | 1 | 0 | Wrong population (>6 months, n=1) | N/A |
| 11 | N/A | PubMed-6 | Backward | 113 | 1 | 0 | Review/editorial (n=1) | N/A |
| 12 | N/A | PubMed-24 | Backward | 136 | 2 | 0 | Duplicate (n=2) | N/A |
| 13 | N/A | PubMed-33 | Backward | 98 | 1 | 0 | Wrong population (>6 months, n=1) | N/A |
| 14 | N/A | PubMed-34 | Backward | 154 | 2 | 0 | Wrong population (>6 months, n=2) | N/A |
| 15 | N/A | PubMed-38 | Backward | 110 | 0 | 0 | N/A | N/A |
| 16 | N/A | PubMed-39 | Backward | 87 | 0 | 0 | N/A | N/A |
| 17 | N/A | PubMed-45 | Backward | 56 | 1 | 0 | Duplicate (n=1) | N/A |
| 18 | N/A | PubMed-51 | Backward | 38 | 1 | 0 | Duplicate (n=1) | N/A |
| 19 | N/A | PubMed-57 | Backward | 49 | 0 | 0 | N/A | N/A |
| 20 | N/A | PubMed-58 | Backward | No data | N/A | N/A | No full text | Full text not retrievable |
| 21 | N/A | PubMed-62 | Backward | 45 | 4 | 0 | Wrong population (>6 months, n=3); Duplicate (n=1) | N/A |
| 65 | 24 Dec 2025 | Backward-30 | Forward | 31 | 1 | 0 | Review/editorial (n=1) | N/A |
| 67 | 26 Dec 2025 | Backward-31 | Forward | 26 | 0 | 0 | N/A | N/A |

This table reports the results of backward and forward citation searches performed on selected source articles to identify additional studies potentially eligible for inclusion. For each round, the table provides the source article ID, date of the search (forward searches only), search type (backward or forward), total results retrieved, number of new articles selected for detailed review, number of articles meeting inclusion criteria, and reasons for exclusion when applicable. N/A indicates that no comment was provided or that the information was not applicable. Exclusion reasons include wrong population (>6 months), duplicate, review/editorial, no PRP intervention, non-autologous intervention, no full text, or other study-specific criteria.
