# Supplementary material for: Platelet-rich plasma for immature post-traumatic scars and early keloids: A scoping review
Source: PLoS One. 2026 Apr 6;21(4):e0345754. doi: 10.1371/journal.pone.0345754 (PMC13052873; doi:10.1371/journal.pone.0345754)
Supplement: S5 Table — This table provides a comprehensive description of the variables used for data extraction in this scoping review. For each variable, definitions, instructions for data entry, and examples are provided to ensure standardized, reproducible, and transparent charting across studies. Variables cover study identification, design, population, PRP intervention specifics, scar characteristics, treatment regimen, outcome measures, follow-up duration, key findings, quantitative efficacy data, adjunct therapies, methodological limitations, adverse events, and additional notes. This dictionary was developed to guide reviewers in systematically extracting relevant data for qualitative synthesis. (DOCX) [file pone.0345754.s008.docx]

# **S5 Table. Data extraction dictionary for charting included studies**

| **Variable** | **Definition / Instructions** | **Example** |
| --- | --- | --- |
| **NO** | Sequential study number for reference in the table | 1, 2, 3… |
| **Author, Year** | Full reference of the study including all authors and year of publication | Kim MJ, Wan J, et al., 2024 |
| **Country** | Country or countries where the study was conducted | Singapore, Hong Kong, Ukraine, Korea |
| **Study Design / Population / PRP Subgroup** | Combine study design (case report, case series, cohort, trial), population description (number, age, sex), and clarification of participants receiving PRP when mixed interventions were applied | Case series; 8 patients: 60 y/o F, 40 y/o F…; PRP subgroup: 3 of 8 patients |
| **Scar Type** | Describe type, location, and characteristics of scars | Post-traumatic, post-operative, chemical burns; facial, breast, body |
| **Time Since Injury** | Time from injury or epithelialization to PRP treatment | ≤6 months post-injury; 7 weeks post-op |
| **ACP/PRP Type** | Specify type of autologous platelet product used | Autologous platelet-rich plasma (activated or non-activated) |
| **PRP Preparation Details** | Describe preparation including centrifugation, platelet concentration, leukocyte content, activation, and other relevant notes | Double centrifugation, 3% calcium chloride activation, leukocyte-rich |
| **Route of Administration** | Delivery method of PRP | Intralesional, topical, intradermal, subcutaneous |
| **Number of Sessions / Dose** | Number of treatment sessions, volume per session, and any dose specifics | 3 sessions at 4-week intervals; 0.1 mL per injection point |
| **Outcome Measures** | Clinical, histological, imaging, or patient-reported outcomes used | VSS, POSAS, dermoscopy, histopathology, photographic documentation |
| **Follow-up Duration** | Duration of patient follow-up after treatment | 6 weeks, 3 months, 12 months; 10 months follow-up |
| **Key Findings** | Main results and observations reported in the study | PRP improved scar appearance, texture, and vascularity; synergistic with botulinum toxin |
| **Quantitative Efficacy Data** | Numeric or statistical outcomes for PRP effects | VSS: 11 → 2; POSAS patient: 49 → 10; 72% of patients achieved >50% POSAS reduction |
| **Combination / Adjunct Therapies** | Any co-interventions used alongside PRP | Rejuran + PRP; PRP combined with fractional CO₂ laser |
| **Study Limitations / Risk of Bias** | Methodological limitations, potential biases, sample size issues, or design concerns | Small sample size; heterogeneous interventions; no control group; retrospective design |
| **Adverse Events** | Any reported adverse events or complications | Mild, transient erythema; no serious adverse events |
| **Comments / Notes** | Additional relevant observations, interpretation, or remarks | Feasibility demonstrated; PRP evidence limited; findings cannot support independent efficacy |

This table provides a comprehensive description of the variables used for data extraction in this scoping review. For each variable, definitions, instructions for data entry, and examples are provided to ensure standardized, reproducible, and transparent charting across studies. Variables cover study identification, design, population, PRP intervention specifics, scar characteristics, treatment regimen, outcome measures, follow-up duration, key findings, quantitative efficacy data, adjunct therapies, methodological limitations, adverse events, and additional notes. This dictionary was developed to guide reviewers in systematically extracting relevant data for qualitative synthesis.
