# Supplementary material for: Platelet-rich plasma for immature post-traumatic scars and early keloids: A scoping review
Source: PLoS One. 2026 Apr 6;21(4):e0345754. doi: 10.1371/journal.pone.0345754 (PMC13052873; doi:10.1371/journal.pone.0345754)
Supplement: S6 Table — This table summarizes the main characteristics, interventions, outcomes, and limitations of studies included in the scoping review, presented in chronological order. Columns provide study reference, country, study design, population characteristics, scar type and timing, PRP type and preparation, route and dosing, outcome measures, follow-up duration, key findings including quantitative efficacy data when available, combination or adjunct therapies, and study limitations including adverse events. Abbreviations used in the table are as follows: PRP, platelet-rich plasma; L-PRP, Leukocyte- and platelet-rich plasma; ACP, autologous conditioned plasma; VSS, Vancouver Scar Scale; POSAS, Patient and Observer Scar Assessment Scale; BTX-A, botulinum toxin type A; FU, follow-up; CaCl₂, calcium chloride; Ca gluconate, calcium gluconate; RCT, randomized controlled trial; F, female; M, male; wks, weeks; mo, months; y, years; intralesional, injection into scar tissue; and topical, applied on scar surface. (DOCX) [file pone.0345754.s009.docx]

# **S6 Table. Chronological summary of included studies with PRP interventions for early and immature scars**

| **Author, Year** | **Country** | **Study Design** | **Population** | **Scar Type** | **Time Since Injury** | **PRP Type** | **PRP Prep & Route** | **PRP Subgroup & Dose** | **Outcome Measures** | **Follow-up** | **Key Findings / Quantitative Data** | **Adjunct Therapies & Notes** | **Limitations / Adverse Events** |
| --- | --- | --- | --- | --- | --- | --- | --- | --- | --- | --- | --- | --- | --- |
| Ruiz A, Cuestas D, et al., 2018 | Colombia / Spain | Case report | 1F, 40 y, Fitzpatrick IV | 2° burn | 7 wks post VASER | L-PRP (autologous)  Platelet concentration: 300–400% above baseline | Manual open technique  Single centrifugation (3200 rpm; 1259 g)  Anticoagulant: sodium citrate.  Activated (10% calcium gluconate; 1:10) | N/A; Topical 6 mL ×5 days/session; Intralesional 6 mL on days (Burn edges) 0,30,60,90,120,180 | VSS  POSAS  Photos | 10 mo | Improved scar appearance, pliability, vascularity, relief, pain, color, irregularity  VSS 11→2  POSAS 49→10 | L-PRP + Single intralesional triamcinolone | Single-patient  Operator-dependent  Limited generalizability  No adverse events |
| Neinaa YM, Elsayed TA, et al., 2021 | Egypt | Single-blind RCT | 60 pts, 12–56 y; 32F,28M | Keloids (head/neck, trunk, extremities) | <6 mo | PRP (autologous)  Leukocyte content: Not specified  Platelet concentration: Not reported | Double centrifugation (first spin at 200 g for 10 minutes; second spin at 1550 g for 10 minutes)  Anticoagulant: sodium citrate  Activation: Not specified  Lower 1/3 plasma  Intralesional | PRP subgroup: 20/60; 3 sessions ×4 wk  Dose: 0.1 mL per injection point  Maximum total volume per session: Not specified | VSS  VRS  Dermoscopy,  Histology, CTGF | ≈12 wk | PRP & BTX-A > TAC; improved vascular pattern  VSS: 85.3±18.3  TAC:46.5±14.3 | None | Small sample  Short follow-up  Single-center  Single-blind  TAC: 20% hypopigmentation |
| Albalat W, Nabil S, et al., 2021 | Egypt | RCT | 160 pts, 20–60 y | Keloids (chronic, multiple sites) | Mean ~4 mo | PRP (autologous)  Leukocyte content: Not specified  Platelet concentration: Not reported | Double centrifugation (First spin: 2000 RBM for 3 minutes; second spin: 5000 RBM for 5 minutes; platelet-containing plasma and buffy coat recentrifuged)  Anticoagulant: sodium citrate  3% CaCl2 activation; Intralesional (30-gauge insulin syringe) | PRP subgroup: 40/160; 6 sessions q3wk  Volume per injection: Not specified  Total volume per session: Not specified | POSAS  Clinical evaluation | 24 wk + 6 wk FU | PRP as effective as triamcinolone; superior to 5-FU  POSAS 92→36  72% achieved >50% reduction | None | Short follow-up  PRP concentration not quantified  No blinding  Heterogeneity  Erythema 35%, mild |
| El-Orabi RA, El-Hadidy MR, et al., 2022 | Egypt | Prospective case series | 15 pts, 2–38 y; 73% M | Immature facial scars (post-trauma/burn) | ≤6 mo | PRP (autologous)  Leukocyte content: Not specified  Platelet concentration: Not reported | Double centrifugation (First spin: 1500 rpm for 10 minutes; second spin: 3000 rpm for 20 minutes)  Blood volume collected: 10 mL peripheral venous blood  Anticoagulant: Sodium citrate  Activation: Not specified  Intralesional (intradermal) | PRP subgroup: 1/15  Number of sessions: Not specified  Interval: Not specified  Dose: Approximately 2 mL per session. | POSAS  Photos | Up to 12 mo | No significant differences  PRP efficacy not assessed | PRP + fractional CO₂ laser + Silicone gel | Very small sample; heterogeneous  No PRP-only group  Subjective outcomes; none reported |
| Kim MJ, Wan J, et al., 2024 | Singapore, HK, Ukraine, Korea | Case series | 8 pts, 14–60 y | Post-trauma/post-op, chemical/iatrogenic; facial, breast, body | 1–6 mo (most ≤3 mo) | PRP (autologous)  Leukocyte content: Not specified  Platelet concentration: Not reported  Enrichment factor: Not reported | Centrifugation method: Not specified  Commercial kit: Not specified  Activation: Not specified  Route of administration: not specified (context suggests injectable use, but route not explicitly stated).  adjunct to Rejuran | PRP subgroup: 3/8  Patients (Cases #4, #5, and #8)  Case #4: Single PRP session  Case #5: 10 PRP sessions  Case #8: 1 PRP session  Interval between sessions: not specified for PRP  Volume/dose per session: Not specified. | Clinical: scar appearance, texture, skin quality  Patient-reported  Photos | 1–3 mo/case | Improved scar appearance, texture  Visual improvements  Synergistic with Rejuran/Botulinum | Rejuran + PRP  Rejuran + BTX  Rejuran + cross-linked HA | Small sample  Heterogeneous scars  No control  Retrospective  Mild transient erythema  No serious adverse events |

This table summarizes the main characteristics, interventions, outcomes, and limitations of studies included in the scoping review, presented in chronological order. Columns provide study reference, country, study design, population characteristics, scar type and timing, PRP type and preparation, route and dosing, outcome measures, follow-up duration, key findings including quantitative efficacy data when available, combination or adjunct therapies, and study limitations including adverse events. Abbreviations used in the table are as follows: PRP, platelet-rich plasma; L-PRP, Leukocyte- and platelet-rich plasma; ACP, autologous conditioned plasma; VSS, Vancouver Scar Scale; POSAS, Patient and Observer Scar Assessment Scale; BTX-A, botulinum toxin type A; FU, follow-up; CaCl₂, calcium chloride; Ca gluconate, calcium gluconate; RCT, randomized controlled trial; F, female; M, male; wks, weeks; mo, months; y, years; intralesional, injection into scar tissue; and topical, applied on scar surface.
