# Supplementary material for: Platelet-rich plasma for immature post-traumatic scars and early keloids: A scoping review
Source: PLoS One. 2026 Apr 6;21(4):e0345754. doi: 10.1371/journal.pone.0345754 (PMC13052873; doi:10.1371/journal.pone.0345754)
Supplement: S7 Table — This table summarizes the critical appraisal of the case report (Aura Ruiz et al., 2024) using the Joanna Briggs Institute (JBI) checklist for case reports. For each domain, “Yes” indicates the criterion was fully addressed, “No” indicates it was not, “Unclear” indicates insufficient information, and “Not applicable” applies when the criterion does not pertain to the study. The checklist evaluates patient demographics, history, clinical presentation, diagnostic assessments, interventions, post-intervention outcomes, adverse events, and clinical lessons. (DOCX) [file pone.0345754.s010.docx]

# **S7 Table. JBI Critical Appraisal Checklist for Case Reports – Ruiz et al., 2024**

| **Question** | **Yes** | **No** | **Unclear** | **Not applicable** |
| --- | --- | --- | --- | --- |
| 1. Were patient’s demographic characteristics clearly described? | ☑ | ☐ | ☐ | ☐ |
| 2. Was the patient’s history clearly described and presented as a timeline? | ☑ | ☐ | ☐ | ☐ |
| 3. Was the current clinical condition of the patient on presentation clearly described? | ☑ | ☐ | ☐ | ☐ |
| 4. Were diagnostic tests or assessment methods and the results clearly described? | ☑ | ☐ | ☐ | ☐ |
| 5. Was the intervention(s) or treatment procedure(s) clearly described? | ☑ | ☐ | ☐ | ☐ |
| 6. Was the post-intervention clinical condition clearly described? | ☑ | ☐ | ☐ | ☐ |
| 7. Were adverse events (harms) or unanticipated events identified and described? | ☐ | ☑ | ☐ | ☐ |
| 8. Does the case report provide takeaway lessons? | ☑ | ☐ | ☐ | ☐ |

This table summarizes the critical appraisal of the case report (Aura Ruiz et al., 2024) using the Joanna Briggs Institute (JBI) checklist for case reports. For each domain, “Yes” indicates the criterion was fully addressed, “No” indicates it was not, “Unclear” indicates insufficient information, and “Not applicable” applies when the criterion does not pertain to the study. The checklist evaluates patient demographics, history, clinical presentation, diagnostic assessments, interventions, post-intervention outcomes, adverse events, and clinical lessons.
