# Supplementary material for: Platelet-rich plasma for immature post-traumatic scars and early keloids: A scoping review
Source: PLoS One. 2026 Apr 6;21(4):e0345754. doi: 10.1371/journal.pone.0345754 (PMC13052873; doi:10.1371/journal.pone.0345754)
Supplement: S8 Table — This checklist assesses methodological quality across ten key domains, including inclusion criteria, measurement and identification of the condition, participant demographics, reporting of clinical information, follow-up outcomes, site characteristics, and appropriateness of statistical analysis. Responses are coded as Yes, No, Unclear, or Not applicable. This assessment is intended to inform evidence mapping and interpretation of study findings in the context of a scoping review. (DOCX) [file pone.0345754.s011.docx]

# **S8 Table. JBI Critical Appraisal of Case Series – Kim et al., 2024**

| **Question** | **Yes** | **No** | **Unclear** | **Not applicable** |
| --- | --- | --- | --- | --- |
| 1. Were there clear criteria for inclusion in the case series? | ☑ | ☐ | ☐ | ☐ |
| 2. Was the condition measured in a standard, reliable way for all participants? | ☐ | ☐ | ☑ | ☐ |
| 3. Were valid methods used for identification of the condition for all participants? | ☑ | ☐ | ☐ | ☐ |
| 4. Did the case series have consecutive inclusion of participants? | ☐ | ☑ | ☐ | ☐ |
| 5. Did the case series have complete inclusion of participants? | ☐ | ☑ | ☐ | ☐ |
| 6. Was there clear reporting of the demographics of the participants? | ☑ | ☐ | ☐ | ☐ |
| 7. Was there clear reporting of clinical information of the participants? | ☑ | ☐ | ☐ | ☐ |
| 8. Were the outcomes or follow-up results of cases clearly reported? | ☑ | ☐ | ☐ | ☐ |
| 9. Was there clear reporting of the presenting site(s)/clinic(s) demographic information? | ☑ | ☐ | ☐ | ☐ |
| 10. Was statistical analysis appropriate? | ☐ | ☑ | ☐ | ☐ |

This checklist assesses methodological quality across ten key domains, including inclusion criteria, measurement and identification of the condition, participant demographics, reporting of clinical information, follow-up outcomes, site characteristics, and appropriateness of statistical analysis. Responses are coded as Yes, No, Unclear, or Not applicable. This assessment is intended to inform evidence mapping and interpretation of study findings in the context of a scoping review.
