# Supplementary material for: Platelet-rich plasma for immature post-traumatic scars and early keloids: A scoping review
Source: PLoS One. 2026 Apr 6;21(4):e0345754. doi: 10.1371/journal.pone.0345754 (PMC13052873; doi:10.1371/journal.pone.0345754)
Supplement: S11 Table — The table presents the seven domains of bias assessed according to the ROBINS-I tool for non-randomized studies: Bias due to confounding, Bias in classification of interventions, Bias in selection of participants, Bias due to missing data, Bias in measurement of outcomes, Bias in selection of the reported result, and the Overall risk of bias. Each domain includes the judgement (Low, Moderate, Serious, or Critical), the key rationale supporting the assessment, and the predicted direction of bias. The predicted direction of bias indicates the potential influence of methodological limitations on study outcomes: “Towards null” suggests an underestimation of treatment effect, “Unpredictable” indicates uncertainty regarding the direction of bias, and “NA” indicates the domain is not applicable. This table provides a transparent overview of methodological strengths and limitations in assessing the comparative efficacy of non-surgical interventions for immature facial scars. (DOCX) [file pone.0345754.s014.docx]

# **S11 Table. ROBINS-I Risk of Bias Assessment – El-Orabi et al., 2022**

| **ROBINS-I domain** | **Judgement** | **Key rationale** | **Predicted direction of bias** |
| --- | --- | --- | --- |
| Bias due to confounding | Critical | Treatment allocation was based on scar characteristics (“strict selection of treatment methods according to the scar characteristics”), resulting in severe confounding by indication. Major prognostic factors—including baseline scar severity, scar age, location, injury mechanism, skin phototype, and genetic predisposition—were neither controlled for in the design nor adjusted for in the analysis. No statistical methods (matching, stratification, multivariable adjustment) were used to address confounding. | Unpredictable |
| Bias in classification of interventions | Moderate | Interventions (silicone gel, corticosteroids, laser, PRP, and combinations) were clearly defined and assigned at baseline. However, adherence—particularly for topical silicone—was not monitored, introducing potential non-differential misclassification that could dilute between-group differences. | Towards null |
| Bias in selection of participants into the study | Low | Follow-up began at treatment initiation, and outcome assessment occurred uniformly at 6 months. There was no evidence of immortal time bias or selection based on post-intervention variables. | NA |
| Bias due to missing data | Low | Outcome (POSAS) and intervention data were complete for all participants, with no reported loss to follow-up. The absence of data on key confounders reflects design limitations rather than missingness among measured variables. | NA |
| Bias in measurement of outcomes | Serious | The primary outcome (POSAS) is subjective and was assessed without blinding. Both patients and observers were aware of the intervention received, creating substantial risk of expectation and observer bias, particularly for more intensive or novel treatments. | Unpredictable |
| Bias in selection of the reported result | Moderate | No pre-registered protocol or pre-specified analysis plan was reported. Multiple outcome measurements derived from POSAS were analyzed, creating potential for selective reporting, although all reported comparisons were non-significant. | Unpredictable |
| Overall risk of bias | Critical | According to the ROBINS-I algorithm, the presence of critical bias due to confounding determines the overall judgment. The study’s comparative conclusion that different non-surgical treatments show “no significant difference” is not a reliable estimate of causal effects. | Unpredictable |

Table presents the seven domains of bias assessed according to the ROBINS-I tool for non-randomized studies: Bias due to confounding, Bias in classification of interventions, Bias in selection of participants, Bias due to missing data, Bias in measurement of outcomes, Bias in selection of the reported result, and the Overall risk of bias. Each domain includes the judgement (Low, Moderate, Serious, or Critical), the key rationale supporting the assessment, and the predicted direction of bias. The predicted direction of bias indicates the potential influence of methodological limitations on study outcomes: “Towards null” suggests an underestimation of treatment effect, “Unpredictable” indicates uncertainty regarding the direction of bias, and “NA” indicates the domain is not applicable. This table provides a transparent overview of methodological strengths and limitations in assessing the comparative efficacy of non-surgical interventions for immature facial scars.
