# Supplementary material for: Platelet-rich plasma for immature post-traumatic scars and early keloids: A scoping review
Source: PLoS One. 2026 Apr 6;21(4):e0345754. doi: 10.1371/journal.pone.0345754 (PMC13052873; doi:10.1371/journal.pone.0345754)
Supplement: S2 Table — This table summarizes the full selection process for studies included in the review. It reports the total number of records retrieved before deduplication, the number of duplicate and unique records identified, and the distribution of exclusions at the title and abstract screening stage. Exclusion reasons are provided according to predefined categories, including population outside the predefined timeframe (>6 months), review/editorial articles, conference abstracts, non–peer-reviewed sources, animal studies, absence of platelet-rich plasma (PRP) intervention, non-autologous PRP, insufficient data, pediatric-only populations, lack of full-text availability, and inappropriate study design. Articles meeting eligibility criteria at this stage were included for full-text review. (DOCX) [file pone.0345754.s005.docx]

# **S2 Table. Summary of article inclusion, deduplication, and exclusion during the selection process.**

| **Category** | **Count** | **Notes** |
| --- | --- | --- |
| Total records retrieved (before deduplication) | 1,325 | Sum of all database search results prior to deduplication |
| Duplicates removed | 79 | Identified during deduplication |
| Unique records after deduplication | 1,246 | Total records screened at title/abstract level |
| Screening exclusions – Wrong population (>6 months) | 972 | E1 |
| Screening exclusions – Review/editorial | 58 | E2 |
| Screening exclusions – Conference abstract | 1 | E3 |
| Screening exclusions – Non-peer-reviewed / Books | 4 | E4 |
| Screening exclusions – Animal study | 143 | E5 |
| Screening exclusions – No PRP intervention | 9 | E6 |
| Screening exclusions – Non-autologous (APC/PRP/other) | 0 | E7 |
| Screening exclusions – Insufficient data | 0 | E8 |
| Screening exclusions – Pediatrics only | 0 | E9 |
| Screening exclusions – Duplicate | 0 | E10 |
| Screening exclusions – No full text | 1 | E11 |
| Screening exclusions – Wrong study design | 25 | E12 |
| Subtotal of excluded after screening | 1,213 | Sum of E1–E12 |
| Included for full-text review | 33 | I |
| Total screened at title/abstract level | 1,246 | Matches unique records after deduplication |

This table summarizes the full selection process for studies included in the review. It reports the total number of records retrieved before deduplication, the number of duplicate and unique records identified, and the distribution of exclusions at the title and abstract screening stage. Exclusion reasons are provided according to predefined categories, including population outside the predefined timeframe (>6 months), review/editorial articles, conference abstracts, non–peer-reviewed sources, animal studies, absence of platelet-rich plasma (PRP) intervention, non-autologous PRP, insufficient data, pediatric-only populations, lack of full-text availability, and inappropriate study design. Articles meeting eligibility criteria at this stage were included for full-text review.
