# Supplementary material for: Platelet-rich plasma for immature post-traumatic scars and early keloids: A scoping review
Source: PLoS One. 2026 Apr 6;21(4):e0345754. doi: 10.1371/journal.pone.0345754 (PMC13052873; doi:10.1371/journal.pone.0345754)
Supplement: S10 Table — The table presents the five domains of bias assessed according to the RoB 2 tool: Domain 1 – randomization process, Domain 2 – deviations from intended interventions, Domain 3 – missing outcome data, Domain 4 – measurement of the outcome, and Domain 5 – selection of the reported result, along with the overall risk of bias judgment. Each domain includes the key signalling questions used to guide assessment and the final judgment (Low risk, Some concerns). The predicted direction of bias indicates the potential influence of methodological limitations on study outcomes, where “Unpredictable” suggests uncertainty regarding the direction of bias. Reviewer comments summarize the rationale for judgments, including randomization, allocation concealment, blinding of participants and assessors, completeness of outcome data, and pre-specification of statistical analyses. Overall, the study shows robust randomization and outcome measurement, but residual concerns arise from lack of participant blinding and per-protocol analysis, resulting in an overall risk of bias rated as Some concerns. (DOCX) [file pone.0345754.s013.docx]

# **S10 Table. Cochrane RoB 2 Assessment – Neinaa et al., 2021**

| **Domain** | **Key signalling question(s)** | **Judgement** |
| --- | --- | --- |
| Domain 1: Randomization process | Was the allocation sequence random and concealed? Were baseline differences balanced? | Low risk |
| Domain 2: Deviations from intended interventions (effect of assignment to intervention) | Were participants and personnel blinded? Were deviations related to awareness of intervention? | Some concerns |
| Domain 3: Missing outcome data | Were outcome data available for all or nearly all participants? | Low risk |
| Domain 4: Measurement of the outcome | Were outcome assessors blinded? Could measurement have been influenced by knowledge of intervention? | Low risk |
| Domain 5: Selection of the reported result | Was the analysis pre-specified? Were multiple outcome measurements or analyses possible? | Some concerns |
| Overall risk of bias | — | Some concerns |

The table presents the five domains of bias assessed according to the RoB 2 tool: Domain 1 – randomization process, Domain 2 – deviations from intended interventions, Domain 3 – missing outcome data, Domain 4 – measurement of the outcome, and Domain 5 – selection of the reported result, along with the overall risk of bias judgment. Each domain includes the key signalling questions used to guide assessment and the final judgment (Low risk, Some concerns). The predicted direction of bias indicates the potential influence of methodological limitations on study outcomes, where “Unpredictable” suggests uncertainty regarding the direction of bias. Reviewer comments summarize the rationale for judgments, including randomization, allocation concealment, blinding of participants and assessors, completeness of outcome data, and pre-specification of statistical analyses. Overall, the study shows robust randomization and outcome measurement, but residual concerns arise from lack of participant blinding and per-protocol analysis, resulting in an overall risk of bias rated as Some concerns.
